# Pharmacokinetics and Pharmacodynamics of Lisdexamfetamine Compared with D-Amphetamine in Healthy Subjects

**DOI:** 10.3389/fphar.2017.00617

**Published:** 2017-09-07

**Authors:** Patrick C. Dolder, Petra Strajhar, Patrick Vizeli, Felix Hammann, Alex Odermatt, Matthias E. Liechti

**Affiliations:** ^1^Division of Clinical Pharmacology and Toxicology, Department of Biomedicine and Department of Clinical Research, University Hospital Basel and University of Basel Basel, Switzerland; ^2^Division of Molecular and Systems Toxicology, Department of Pharmaceutical Sciences, University of Basel Basel, Switzerland

**Keywords:** D-amphetamine, lisdexamfetamine, subjective effects, autonomic effects, healthy subjects

## Abstract

**Rationale:** Lisdexamfetamine is a prodrug of D-amphetamine used for the treatment of attention-deficit/hyperactivity disorder (ADHD). Lisdexamfetamine is thought to have a prolonged pharmacokinetic profile compared with oral D-amphetamine, possibly associated with lower drug liking and a lower risk of oral misuse. However, differences in the pharmacokinetics and pharmacodynamics of lisdexamfetamine and D-amphetamine have not been directly compared.

**Methods:** Equimolar doses of D-amphetamine (40 mg) and lisdexamfetamine (100 mg), and placebo were administered in 24 healthy subjects in a randomized, double-blind, placebo-controlled, cross-over study. Plasma concentrations of amphetamine, subjective effects, and vital signs were repeatedly assessed. The pharmacokinetic parameters were determined using compartmental modeling.

**Results:** The increase in plasma concentrations of amphetamine had a 0.6 ± 0.6 h (mean ± SD) longer lag time and reached peak levels 1.1 ± 1.5 h later after lisdexamfetamine administration compared with D-amphetamine administration, but no differences in maximal concentrations or total exposure (AUC) were found between the two treatments. Consistent with the pharmacokinetics, the subjective and cardiovascular stimulant effects of lisdexamfetamine also occurred later compared with D-amphetamine. However, no differences in peak ratings of potentially abuse-related subjective drug effects (e.g., drug liking, drug high, stimulation, happy, well-being, and self-confidence) were observed after lisdexamfetamine administration compared with D-amphetamine administration. Lisdexamfetamine and D-amphetamine also produced similar peak increases in mean arterial blood pressure, heart rate, body temperature, pupil size, and adverse effects.

**Conclusion:** The pharmacokinetics and pharmacodynamics of lisdexamfetamine are similar to D-amphetamine administered 1h later. Lisdexamfetamine is likely associated with a similar risk of oral abuse as D-amphetamine. The study was registered at ClinicalTrials.gov (NCT02668926).

## Introduction

Lisdexamfetamine is an inactive prodrug formulation of D-amphetamine (Krishnan and Stark, 2008; Krishnan et al., 2008; Hutson et al., 2014) that is marketed for the treatment of attention-deficit/hyperactivity disorder (ADHD). D-amphetamine is used as a second-line treatment for ADHD. D-amphetamine is similarly or even more effective than methylphenidate in the treatment of ADHD (Faraone and Buitelaar, 2010). However, amphetamine is also misused recreationally to induce euphoria or enhance cognitive performance, with a lifetime use prevalence of 5.5–15% in adults (EMCDDA, 2016; Johnston et al., 2016). Lisdexamfetamine is thought to have lower abuse potential than D-amphetamine (Heal et al., 2013). Inactive lisdexamfetamine is completely (>98%) converted to its active metabolite D-amphetamine in the circulation (Pennick, 2010; Sharman and Pennick, 2014). When lisdexamfetamine is misused intranasally or intravenously, the pharmacokinetics are similar to oral use (Jasinski and Krishnan, 2009b; Ermer et al., 2011), and the subjective effects are not enhanced by parenteral administration in contrast to D-amphetamine (Lile et al., 2011) thus reducing the risk of parenteral misuse of lisdexamfetamine compared with D-amphetamine. Intravenous lisdexamfetamine use also produced significantly lower increases in “drug liking” and “stimulant effects” compared with D-amphetamine in intravenous substance users (Jasinski and Krishnan, 2009a). After oral administration, the conversion of lisdexamfetamine to D-amphetamine is thought to occur gradually, reportedly resulting in a prolonged pharmacokinetic profile with low peak but sustained plasma amphetamine concentrations (Jasinski and Krishnan, 2009a; Steer et al., 2012). Such a prolonged pharmacokinetic profile is considered to be associated with slower effects on dopamine release, lower euphoric effects, and a possibly lower risk of misuse (Jasinski and Krishnan, 2009a; Heal et al., 2013; Coghill et al., 2014). This view is supported by animal studies. In rats, the peak plasma concentration (*C*_max_) of amphetamine was lower after lisdexamfetamine, and it produced a gradual and sustained increase in dopamine efflux and much less locomotor activity compared with D-amphetamine (Rowley et al., 2012). Thus, in this rat study, lisdexamfetamine was shown to have markedly less stimulant effects than an equivalent dose of D-amphetamine, with both drugs administered intraperitoneally (Rowley et al., 2012). However, differences in the pharmacokinetics of lisdexamfetamine and D-amphetamine after oral administration have not been studied in humans. Additionally, we are aware of only one study that directly compared the acute pharmacodynamic effects of lisdexamfetamine and D-amphetamine within-subjects in humans (Jasinski and Krishnan, 2009a). In current stimulant users, 100 mg lisdexamfetamine produced significantly lower subjective “drug liking” than an equivalent dose of 40 mg D-amphetamine (Jasinski and Krishnan, 2009a). However, subjective drug effects were comparable on the morphine-benzedrine scale (euphoria), amphetamine scale, and benzedrine (stimulation) scale of the Addiction Research Center Inventory (ARCI) (Jasinski and Krishnan, 2009a). Nonetheless, this previous study (Jasinski and Krishnan, 2009a) did not assess the pharmacokinetics of amphetamine to demonstrate the equivalence of the doses used. Additionally, data from healthy non-stimulant-using subjects are lacking, and no industry-independent studies have been conducted. Therefore, in the present study, we directly compared both pharmacokinetic and pharmacodynamic differences between equimolar oral doses of lisdexamfetamine and D-amphetamine in healthy, non-stimulant-using subjects. Based on data from animal studies (Rowley et al., 2012) and limited human data (Jasinski and Krishnan, 2009a), we hypothesized that lisdexamfetamine would have (i) a longer time to *C*_max_ (*T*_max_) than D-amphetamine, (ii) a lower *C*_max_ than D-amphetamine, (iii) an area under the amphetamine concentration-time curve that is identical to D-amphetamine, (iv) a smaller maximal effect (*E*_max_) and (v) a longer time to *E*_max_ (*T*_max_) than D-amphetamine, and (vi) an area under the observed subjective drug effect-time curve that is identical to D-amphetamine.

## Methods

### Study Design

The present study used a double-blind, placebo-controlled, cross-over design with three experimental test days (D-amphetamine, lisdexamfetamine, and placebo) in balanced order. The washout periods between sessions were at least 7 days. The study was conducted in accordance with the Declaration of Helsinki and International Conference on Harmonization Guidelines in Good Clinical Practice and approved by the Ethics Committee northwest/central Switzerland (EKNZ) and Swiss Agency for Therapeutic Products (Swissmedic). All of the subjects provided written consent before participating in the study, and they were paid for their participation. The study was registered at ClinicalTrials.gov (NCT02668926).

### Participants

Twenty-four healthy subjects (12 men, 12 women) with a mean ± SD age of 25.3 ± 3.0 years (range: 21–34 years) were recruited from the University of Basel. Inclusion criteria were age 18–45 years, body mass index 18–27 kg/m^2^, and birth control for women. Subjects with a personal or first-degree-relative history of psychiatric disorders or chronic or acute physical illness were excluded. Additional exclusion criteria were tobacco smoking (>10 cigarettes/day), the consumption of alcoholic drinks (>10/week), and a lifetime history of using illicit drugs more than five times, with the exception of occasional cannabis use in the past. Subjects who used any illicit drugs, including cannabis, within the past 2 months or during the study period were excluded. The subjects were asked to abstain from excessive alcohol consumption between test sessions and not to drink caffeine-containing liquids after midnight before the study day. We performed drug tests at screening and before each test session using TRIAGE 8 (Biosite, San Diego, CA, United States). Female subjects were investigated during the follicular phase of their menstrual cycle (days 2–14) to account for cyclic changes in the reactivity to D-amphetamine (White et al., 2002).

### Study Procedures

The study included a screening visit, three experimental sessions (test days), and an end-of-study visit. Experimental sessions began at 8:00 AM. An indwelling intravenous catheter was placed in an antecubital vein for blood sampling. A single oral dose of D-amphetamine, lisdexamfetamine, or placebo was administered at 9:00 AM. Autonomic and subjective drug effects were assessed repeatedly throughout the session. For the analysis of amphetamine concentrations in plasma, blood samples were collected in lithium heparin tubes 1 h before and 0, 0.5, 1, 1.5, 2, 2.5, 3, 3.5, 4, 5, 6, 8, 10, 12, and 24 h after drug administration. The blood samples were immediately centrifuged, and the plasma was rapidly stored at -20°C and later at -80°C until analysis. During the test sessions, the subjects did not engage in any physical activity, were resting in hospital beds in a calm standard hospital room, and were served a standardized lunch and dinner at 11:30 AM and 6:30 PM, respectively. The test session ended at 9:00 PM. The subjects returned home and returned the following day at 9:00 AM for the final 24 h measurements and drawing of blood samples.

### Study Drugs

Gelatin capsules that contained either lisdexamfetamine dimesylate (100 mg salt; Opopharma, Rümlang, Switzerland) or D-amphetamine sulfate (40.3 mg salt; Hänseler, Herisau, Switzerland), both corresponding to an equivalent dose of 29.6 mg D-amphetamine, and placebo capsules (mannitol) were prepared by the pharmacy of the University Hospital Basel according to Good Manufacturing Practice. D-amphetamine and placebo (Mannitol) were first encapsulated using size 3 gelatine capsules, similar to the marketed lisdexamfetamine capsules. Then all capsules were additionally encabulated using opaque size AA gelatin capsules to ensure blinding. Subjects ingested the capsules together with tap water. To induce greater subjective drug liking and mimic misuse, the selected dose of lisdexamfetamine was relatively high and above the upper recommended daily dose of 70 mg.

### Measures

#### Quantification of Amphetamine Concentrations in Blood Plasma

Plasma concentrations of amphetamine were measured by ultra-high pressure liquid chromatography-mass spectrometry/mass spectrometry. The materials, procedures, and method validation are described in detail in the Supplementary Material (Supplementary Methods). Lower limits of detection and quantification were 0.26 and 0.78 ng/ml, respectively. Concentration profiles of lisdexamfetamine were previously shown (Ermer et al., 2010) and were not determined in the present study.

#### Subjective Effects

Visual Analog Scales (VASs) were repeatedly used to assess subjective effects over time. The VASs included “any drug effect,” “good drug effect,” “bad drug effect,” “drug liking,” “drug high,” “stimulated,” “alertness,” “content,” “happy,” “closeness to others,” “talkative,” “open,” “concentration,” “trust,” and “want to be with others” and have previously been used (Schmid et al., 2014, 2015b). The VASs were presented as 100-mm horizontal lines (0 to +100), marked from “not at all” on the left to “extremely” on the right. The VASs for “happy,” “closeness to others,” “open,” “trust,” and “I want to be with others” were bidirectional (±50), marked from “not at all” on the left (-50), to “normal” in the middle (0), to “extremely” on the right (+50). The VASs were administered 1 h before and 0, 0.5, 1, 1.5, 2, 2.5, 3, 3.5, 4, 5, 6, 7, 8, 9, 10, 11, 12, and 24 h after drug administration.

The 60-item Adjective Mood Rating Scale (AMRS) (Janke and Debus, 1978) was administered 1 h before and 2, 3, 4, 12, and 24 h after drug administration. The AMRS subscales for well-being, extroversion, emotional excitability, and self-confidence have previously been shown to be sensitive to the effects of psychostimulants (Hysek et al., 2014; Schmid et al., 2015a).

#### Autonomic Effects

Blood pressure, heart rate, and tympanic body temperature were repeatedly measured 1 h before and 0, 0.5, 1, 1.5, 2.0, 2.5, 3, 3.5, 4, 5, 6, 7, 8, 9, 10, 11, 12, and 24 h after drug administration. Diastolic and systolic blood pressure and heart rate were measured using an automatic oscillometric device (OMRON Healthcare Europe NA, Hoofddorp, Netherlands). The measurements were performed in duplicate at an interval of 1 min and after a resting time of at least 10 min. The averages were calculated for analysis. The rate-pressure product was calculated as systolic blood pressure × heart rate. Core (tympanic) temperature was measured using an GENIUSTM 2 ear thermometer (Tyco Healthcare Group LP, Watertown, NY, United States). Pupillometry was performed 1 h before and 0, 0.5, 1, 1.5, 2, 2.5, 3, 3.5, 4, 5, 6, 8, 10, 12, and 24 h after drug administration using a hand-held PRL 200 infrared pupillometer (NeurOptics, Irvine, CA, United States). Pupil function was measured under standardized dark-light conditions and assessed by a Voltcraft MS-1300 luxmeter (Voltcraft, Hirschau, Germany) following a dark adaptation time of 1 min as previously described (Hysek and Liechti, 2012).

#### Adverse Effects

Adverse effects were assessed 1 h before and 12 h (acute) and 24 h (sub-acute) after drug administration using the 66-item List of Complaints (Zerssen, 1976). The scale yields a total adverse effects score and reliably measures physical and general discomfort.

### Pharmacokinetic and Exposure Effect Relationship Analyses

All of the pharmacokinetic and pharmacodynamic analyses were performed using Phoenix WinNonlin 6.4 (Certara, Princeton, NJ, United States). Pharmacokinetic parameters were estimated using compartmental modeling. A one-compartment model was used with first-order input, first-order elimination, and lag time. Initial estimates were derived from non-compartmental analyses. The model fit was assessed by visual inspection and Akaike information criteria. The model fit was impaired without lag time and not relevantly improved by a two-compartment model. A non-compartmental analysis was also performed prior to the modeling. Peak plasma concentration (*C*_max_) and time to *C*_max_ (*T*_max_) were obtained directly from the observed data. The terminal elimination rate constant (λ*_z_*) was estimated by log-linear regression after semi-logarithmic transformation of the data using at least three data points of the terminal linear phase of the concentration-time curve. The area under the concentration-time curve (AUC) from 0 to 24 h after dosing (AUC_24_) was calculated using the trapezoidal method. The AUC to infinity (AUC_∞_) was determined by extrapolation of the AUC_24_ by using λ*_z_*.

The lisdexamfetamine- and D-amphetamine-induced subjective and autonomic effects were determined as differences from placebo in the same subject at corresponding time points to control for circadian changes and placebo effects (Supplementary Table S2). Maximal effect (*E*_max_) and the time to reach *E*_max_ (*T*_max_) of the pharmacodynamic response were determined directly from the observed effect-time curves. The area under the observed effect-time curve (AUEC) was determined using the trapezoidal method. The onset of the response was determined using the effect-time curve, with 10% of the individual maximal response as the threshold. To assess the amphetamine exposure-effect relationship, the changes in pharmacodynamic effect after lisdexamfetamine and D-amphetamine administration for each time point were plotted against the respective plasma concentrations of amphetamine (hysteresis plots).

### Statistical Analyses

The data were analyzed using repeated-measures analysis of variance (ANOVA), with drug as the within-subjects factor. Repeated measures are expressed as *E*_max_ and AUEC values prior to the ANOVA. Tukey *post hoc* comparisons were performed based on significant main effects of drug. Plasma amphetamine concentrations after administration of lisdexamfetamine- and D-amphetamine and differences from placebo were compared using paired *t*-tests. Sex differences were assessed by adding sex as additional between-subject factor to the ANOVAs. The criterion for significance was *p* < 0.05.

## Results

### Pharmacokinetics

**FIGURE 1:**
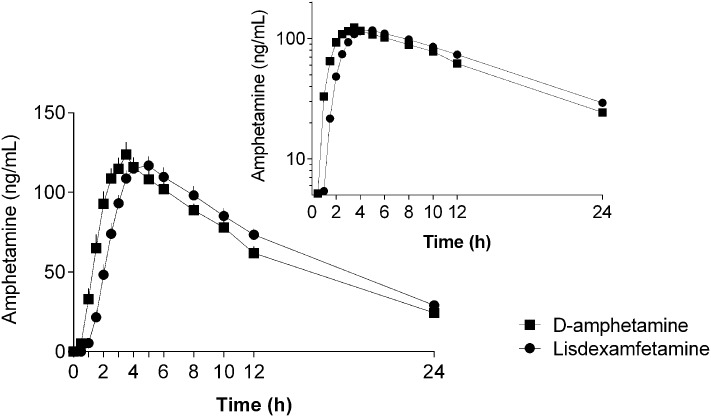
Amphetamine concentration-time curves (mean ± SEM) in 24 and 23 subjects after administration of lisdexamfetamine and D-amphetamine, respectively. The onset and peak times of the amphetamine concentration-time curve were longer after lisdexamfetamine administration compared with D-amphetamine administration, but no differences were found in the maximal concentrations, areas under the concentration-time curves, or absorption or elimination constants between the two treatments. The inset shows the semilogarithmic plot. The amphetamine concentration-time curves were shifted to the right after lisdexamfetamine administration compared with D-amphetamine administration but were otherwise almost identical. The drugs were administered at *t* = 0. The corresponding pharmacokinetic parameters were derived from compartmental and non-compartmental analyses and are shown in **Table 1** and Supplementary Table S1, respectively.

The plasma amphetamine concentration-time curves after D-amphetamine and lisdexamfetamine administration are shown in **Figure 1**. Individual plots are shown in **Supplementary Figure S1**. The intravenous catheter could not be placed and plasma was not collected in one subject after D-amphetamine administration. The corresponding pharmacokinetic parameters that were derived from the compartmental and non-compartmental analyses are shown in **Table 1** and Supplementary Table S1, respectively. As planned, the administration of equimolar doses of D-amphetamine and lisdexamfetamine resulted in similar AUC values. The increase in plasma amphetamine concentrations had a 0.6 ± 0.6 h (mean ± SD) longer lag time and reached peak levels 1.1 ± 1.5 h later after lisdexamfetamine administration compared with D-amphetamine administration (**Figure 1**, **Table 1**, and Supplementary Table S1). Both *T*_lag_ and *T*_max_ values were significantly different (*t* = 2.87, *p* < 0.001, and *t* = 3.54, *p* < 0.001, respectively; **Table 1**) between the two active drug conditions. However, the absorption constant, *K*_01_, was only non-significantly greater after D-amphetamine administration compared with lisdexamfetamine administration (*t* = 1.86, *p* = 0.07). *C*_max_ values were similar (**Table 1** and Supplementary Table S1) after the administration of both drugs. Thus, in contrast to our hypothesis, a curve shift was observed, but no relevant difference in the shape or peak size of the two amphetamine concentration-time curves was found (**Figure 1**). There were no differences in the pharmacokinetics of lisdexamfetamine or D-amphetamine between men and women.

**Table 1:** Pharmacokinetic parameters for amphetamine based on compartmental modeling.

Dose	*N*	*k*_01_ (1/h)	λ (1/h)	*V*_d_ (L)	*C*_max_ (ng/mL)	*t*_lag_	*t*_max_ (h)	*t*_1/2_ (h)	AUC_∞_ (ng ⋅ h/ml_)	CL/F (L/h)
D-amphetamine	23 geometric mean (95% CI)	1.3 (0.84–1.95)	0.088 (0.077–0.101)	195 (172–220)	120 (108–133)	0.8 (0.6–1.0)	3.3 (2.7–3.9)	7.9 (6.9–9.1)	1727 (1540–1935)	17 (15–19)
	Range	0.41–17	0.046–0.162	113–375	77–181	0.3–2.0	0.9–5.9	4.3–15	1116–3463	9–27
Lisdexamfetamine	24 geometric mean (95% CI)	0.78 (0.63–0.98)	0.088 (0.078–0.098)	186 (166–209)	118 (108–128)	1.5 (1.3–1.7)^∗∗∗^	4.6 (4.1–5.2)^∗∗∗^	7.9 (7.1–8.9)	1817 (1637–2017)	16 (15–18)
	Range	0.25–1.9	0.055–0.148	88–266	83–174	0.8–2.4	2.5–8.4	4.7–13	1087–3031	10–27


*AUC_*8*_, area under the plasma concentration-time curve from time zero to infinity; *C*_*max*_, estimated maximum plasma concentration; *t*_*1/2*_, estimated plasma elimination half -life; *t*_*lag*_, lag time or time; *t*_*max*_, estimated time to reach *C*_*max*_; *k*_*01*_, first-order absorption coefficient; λ, first order elimination coefficient; *V*_*d*_, volume of distribution. ^∗∗∗^*P* < 0.001 compared with D-amphetamine (two-sided *T*-tests).*

### Subjective Effects

**FIGURE 2:**
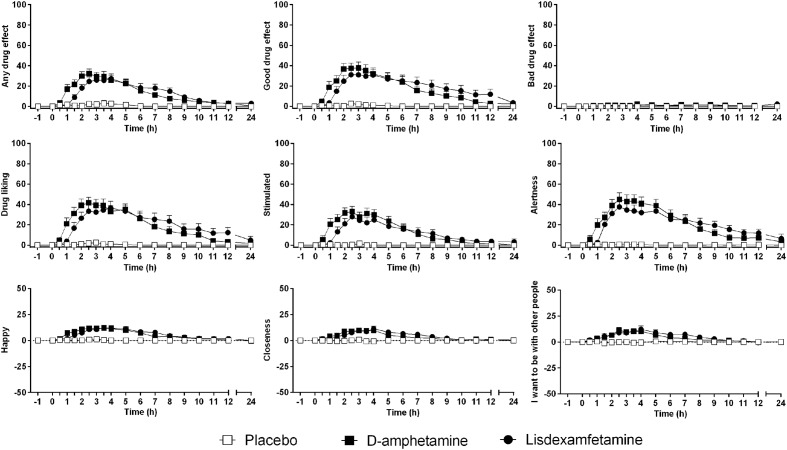
Lisdexamfetamine and D-amphetamine produced similar subjective responses compared with placebo. The effect onset and maximal response were non-significantly delayed after lisdexamfetamine administration compared with D-amphetamine administration, but the maximal effects and curve shapes were similar. The data are expressed as the mean ± SEM in 24 subjects.

Subjective drug effects over time are shown in **Figure 2**. Lisdexamfetamine and D-amphetamine produced similar increases in VAS and AMRS scores compared with placebo (**Figure 2** and **Supplementary Figure S2**, respectively; **Table 1** and Supplementary Table S2). The subjective drug effect-time curves were shifted to the right consistent with significantly longer *T*_onset_ and *T*_max_ values after lisdexamfetamine administration compared with D-amphetamine administration, consistent with the pharmacokinetics of the two drugs. However, no differences in *E*_max_ or AUEC values were found between lisdexamfetamine and D-amphetamine. After both lisdexamfetamine and D-amphetamine administration, the subjective drug effect-concentration curves revealed similar clockwise hysteresis, indicating similar extents of acute pharmacological tolerance to lisdexamfetamine and D-amphetamine (**Supplementary Figure S3**). Sex did not moderate the subjective effects after lisdexamfetamine and D-amphetamine.

### Autonomic Effects

**FIGURE 3:**
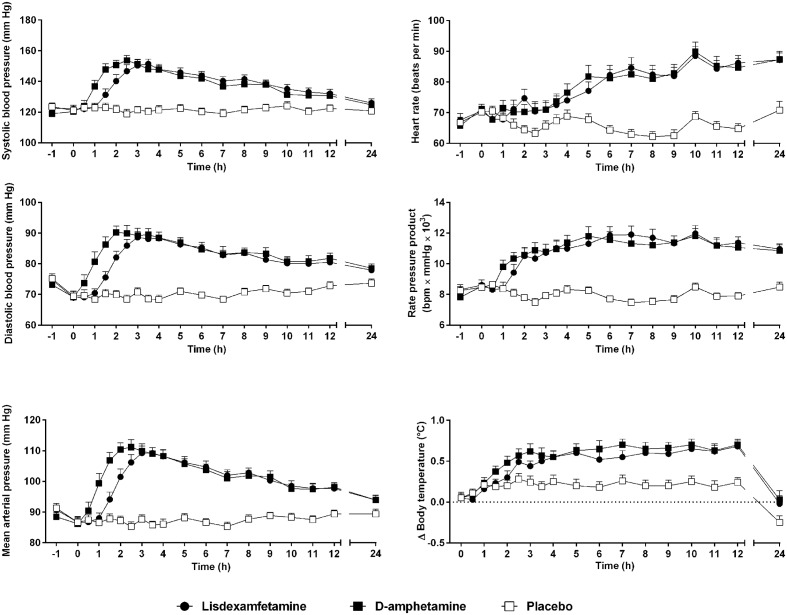
Lisdexamfetamine and D-amphetamine produced similar cardiostimulant responses compared with placebo. The blood pressure response onset was delayed and the diastolic pressure response was reduced after lisdexamfetamine administration compared with D-amphetamine administration. However, the rate-pressure product, reflecting the overall cardiovascular response, similarly increased after both active treatments compared with placebo. The data are expressed as the mean ± SEM in 24 subjects.

Vital signs over time are shown in **Figure 3**. Lisdexamfetamine and D-amphetamine produced similar increases in blood pressure, heart rate, body temperature, and pupil size (**Figure 3**, **Table 2**, **Supplementary Figure S4** and Table S2). The blood pressure-time curves were shifted to the right because of significantly longer *T*_onset_ values after lisdexamfetamine administration compared with D-amphetamine administration (**Figure 3** and Supplementary Table S2). Diastolic blood pressure reached significantly higher values after D-amphetamine administration compared with lisdexamfetamine administration (**Table 2**). No differences were found in the placebo-adjusted increases in diastolic blood pressure (Supplementary Table S2), mean arterial pressure, or rate-pressure product (systolic blood pressure × heart rate), indicating similar overall cardiovascular stimulant effects after the two treatments (**Table 2**). After both lisdexamfetamine and D-amphetamine administration, the blood pressure responses returned to baseline faster than the plasma levels of amphetamine (**Figures 1**, **3**), whereas the heart rate responses increased more slowly and remained high up to 24 h. The blood pressure-concentration plot presented clockwise hysteresis, similar to the subjective drug effect-concentration plots, indicating acute pharmacological tolerance (**Supplementary Figure S5**). In contrast, the heart rate responses presented counterclockwise hysteresis in the effect-concentration plots, indicating that the responses lagged behind the changes in plasma concentration, with no tolerance (**Supplementary Figure S5**) to the effects of either lisdexamfetamine or D-amphetamine. These results indicate that there were no differences in the effect-concentration relationships between lisdexamfetamine and D-amphetamine. Finally, sex had no influence on the cardiovascular effects of the compounds.

**Table 2:** Comparison of the maximal pharmacodynamic effects of lisdexamfetamine and D-amphetamine.

		Placebo (mean ± SEM)	Lisdexamfetamine (mean ± SEM)	D-amphetamine (mean ± SEM)	Main effect of drug *F*_2,46_
**Autonomic effects**					
Systolic blood pressure (mmHg)	*E*_max_	131 ± 2.7	157 ± 3.1***	158 ± 2.8***	106.56
Diastolic blood pressure (mmHg)	*E*_max_	79 ± 1.1	93 ± 1.7***	97 ± 1.8***#	93.97
Mean arterial blood pressure (mmHg)	*E*_max_	96 ± 1.2	114 ± 2.0***	116 ± 1.8***	111.47
Heart rate (beats/min)	*E*_max_	76 ± 1.5	94 ± 3.1***	94 ± 3.4***	28.42
Rate pressure product (beats mmHg/min	*E*_max_	9655 ± 236	13083 ± 561***	13245 ± 603***	43.01
Body temperature (°C)	*E*_max_	37.3 ± 0.07	37.7 ± 0.06***	37.7 ± 0.07***	24.05
Pupil size (mm)	*E*_max_	6.8 ± 0.09	7.4 ± 0.11***	7.4 ± 0.10***	79.56
Pupil size after light stimulus (mm)	*E*_max_	5.0 ± 0.08	5.8 ± 0.11***	5.7 ± 0.11***	55.46
Constriction amplitude (mm)	*E*_min_	1.7 ± 0.04	1.5 ± 0.08	1.6 ± 0.05	5.03
**Subjective effects**					
**Visual Analog Scale (VAS, %max)**					
Any drug effect	*E*_max_	5.3 ± 3.1	36 ± 4.9***	39 ± 4.8***	27.31
Good drug effect	*E*_max_	4.0 ± 2.5	42 ± 6.5***	49 ± 5.6***	36.65
Bad drug effect	*E*_max_	0.04 ± 0.04	5.1 ± 1.9*	4.0 ± 1.3	4.81
Drug liking	*E*_max_	3.7 ± 2.6	48 ± 6.9***	51 ± 5.8***	37.57
Drug high	*E*_max_	3.3 ± 2.6	29 ± 6.3***	36 ± 5.6***	16.98
Stimulated	*E*_max_	2.4 ± 1.7	38 ± 6.9***	44 ± 5.7***	24.97
Alertness	*E*_max_	2.4 ± 1.1	50 ± 7.2***	56 ± 6.4***	41.31
Content	*E*_max_	1.1 ± 0.63	19 ± 3.0***	18 ± 2.9***	24.93
Happy	*E*_max_	1.7 ± 1.3	18 ± 2.9***	17 ± 2.8***	21.77
Closeness to others	*E*_max_	0.79 ± 0.79	15 ± 2.8***	15 ± 2.5***	19.01
Talkative	*E*_max_	1.3 ± 1.0	23 ± 2.7***	21 ± 2.3***	39.33
Open	*E*_max_	0.88 ± 0.79	22 ± 2.6***	22 ± 2.7***	43.55
Concentration	*E*_max_	0.38 ± 0.27	21 ± 3.1***	16 ± 2.8***	26.97
Trust	*E*_max_	0.96 ± 0.96	15 ± 2.6***	17 ± 3.1***	21.18
I want to be with others	*E*_max_	1.3 ± 0.89	18 ± 3.6***	16 ± 3.1***	15.60
**Adjective Mood Rating Scale (AMRS score)**					
Well-being	Δ*E*_max_	1.9 ± 0.7	4.6 ± 0.6**	5.6 ± 0.6***	11.48
Extroversion	Δ*E*_max_	1.3 ± 0.4	3.4 ± 0.4***	3.4 ± 0.4***	13.61
Excitability	Δ*E*_max_	0 ± 0.2	1.6 ± 0.5**	2.4 ± 0.4***	14.58
Self-confidence	Δ*E*_max_	0.9 ± 0.3	2.2 ± 0.4*	2.8 ± 0.5**	6.72
**Adverse Effects**					
Acute adverse effects	Δ12 h	-0.3 ± 0.3	6.6 ± 1.0***	6.7 ± 0.9***	29.25
Sub-acute adverse effects	Δ24 h	-0.8 ± 0.3	7.3 ± 1.5***	6.9 ± 1.2***	26.72


*Values are mean ± SEM in 24 subjects. ^∗^*P* < 0.05, ^∗∗^*P* < 0.01, ^∗∗∗^*P* < 0.001 compared with placebo. ^#^*P* < 0.05 compared with lisdexamfetamine.*

### Adverse Effects

Both lisdexamfetamine and D-amphetamine increased acute and subacute adverse effect ratings compared with placebo (**Table 2**). Acute adverse effects mainly included a lack of appetite and dry mouth in most of the subjects. Subacute adverse effects mainly included insomnia in most of the subjects after both treatments.

## Discussion

The present study compared the pharmacokinetics and pharmacodynamics of lisdexamfetamine and D-amphetamine within-subjects in healthy volunteers. In contrast to our hypothesis, no differences were found in the peak plasma concentrations of amphetamine and the associated subjective and cardiovascular peak effects between lisdexamfetamine and D-amphetamine. Increases in the plasma concentrations of amphetamine occurred an average of 0.6 h later and reached peak levels 1.1 h later after lisdexamfetamine administration compared with D-amphetamine administration, but the amphetamine concentration-time and drug effect-time curves were otherwise comparable between treatments. Thus, the pharmacokinetics and pharmacodynamics of a high dose of the newly marketed medication lisdexamfetamine were practically identical to an equimolar dose of the classic immediate-release D-amphetamine administered 1 h later. The present data indicate that the conversion of the prodrug lisdexamfetamine to D-amphetamine slightly delays the onset of the increase in amphetamine concentrations in the body without causing relevant alterations in the slope or maximal concentrations.

Pharmacokinetic factors, such as rapid drug delivery to the brain, are important predictors of abuse liability (Busto and Sellers, 1986; Volkow and Swanson, 2003; Smith et al., 2016). Substances with a slow absorption rate are less likely to be abused than drugs with a rapid absorption rate (Busto and Sellers, 1986; Farre and Cami, 1991; Volkow and Swanson, 2003). A slow rise of simulant blood concentration, which is usually observed with extended-release formulations, is associated with lower subjective effects and possibly lower abuse potential (Parasrampuria et al., 2007; Smith et al., 2016). Lisdexamfetamine was reportedly developed with the goal of providing a long duration of action and lower abuse potential (Jasinski and Krishnan, 2009a; Steer et al., 2012). Preliminary unpublished data that were reported in a previous study (Jasinski and Krishnan, 2009a) indicated a longer *T*_max_ and lower *C*_max_ of D-amphetamine following lisdexamfetamine compared with D-amphetamine administration. However, the present study found no such difference in *C*_max_ values after lisdexamfetamine and D-amphetamine administration. A previous study compared the pharmacodynamics (but not pharmacokinetics) of lisdexamfetamine and D-amphetamine and found lower peak ratings of drug liking in current stimulant users after lisdexamfetamine administration compared with D-amphetamine administration using the same doses as in the present study (Jasinski and Krishnan, 2009a). However, ratings of drug liking in the stimulant users reached mean peak levels that were only 17% of the scale maximum after administration of 40 mg D-amphetamine (Jasinski and Krishnan, 2009a). In the present study, mean ratings reached 51 and 48% of peak scale levels in the healthy and mostly stimulant-naive subjects after D-amphetamine and lisdexamfetamine administration, respectively. Additionally, lisdexamfetamine and D-amphetamine produced similar peak euphoria and amphetamine effects on the ARCI and cardiovascular effects and were reported by stimulant users to have similar abuse-related monetary street value (Jasinski and Krishnan, 2009a). These latter findings in stimulant users are consistent with our results, in which we found no relevant differences between the pharmacokinetics and pharmacodynamics of lisdexamfetamine and D-amphetamine. A study in adults with ADHD also reported comparable cardiovascular stimulation after administration of 50 mg lisdexamfetamine and 20 mg of mixed immediate-release amphetamine salts (Martin et al., 2014). An analysis of exposures that were reported to poison centers reported overall similar clinical effects of lisdexamfetamine and D-amphetamine, including agitation, tachycardia, and hypertension (Kaland and Klein-Schwartz, 2015). A marked increase in reported lisdexamfetamine misuse cases was reported to poison centers between 2007 and 2012, resulting in more cases associated with lisdexamfetamine than extended-release D-amphetamine (Kaland and Klein-Schwartz, 2015).

Intranasal and intravenous lisdexamfetamine use has been shown to result in delayed and reduced subjective effects (Jasinski and Krishnan, 2009b; Ermer et al., 2011). In contrast, the minimal changes in the oral pharmacokinetics and pharmacodynamics of lisdexamfetamine compared with D-amphetamine that were observed in the present study did not relevantly slow the rise of amphetamine concentrations or subjective effects and thus were not sufficient to reduce the abuse potential of oral lisdexamfetamine use. In contrast, clear differences were found between the kinetics of extended-release and immediate-release formulations (Clausen et al., 2005; Parasrampuria et al., 2007) and possibly also between extended-release formulations and lisdexamfetamine (Rosen et al., 1965; Haffey et al., 2009).

The present study has limitations. We used only one relatively high dose of lisdexamfetamine and D-amphetamine. We cannot exclude possible differences in the pharmacokinetics and pharmacodynamics of lisdexamfetamine and D-amphetamine at lower or higher doses than those used in the present study. Additional studies that administer 50 and 150 mg lisdexamfetamine and 20 and 60 mg D-amphetamine, respectively, would be needed to further validate the present findings. The recommended doses of lisdexamfetamine for the treatment of ADHD are 30–70 mg/day, with an initial dose of 30 mg. Thus, the present study used a higher single dose (100 mg) in non-treated subjects to mimic the misuse of lisdexamfetamine and to produce similar plasma concentrations after a single dose to those reached during repeated administration of 70 mg when steady state is reached. Additionally, our subjects were fasted when the drugs were administered. *T*_max_ values have been reported to be prolonged by approximately 1 h in the fed state compared with the fasted state (Krishnan and Zhang, 2008). Furthermore, repeated lisdexamfetamine administration results in tolerance to the pronounced subjective and cardiostimulant effects, which has been reported with chronic use (Adler et al., 2009b; Findling et al., 2011; Steer et al., 2012). Similarly, acute insomnia was observed in the majority of the subjects after the single high-dose administration of lisdexamfetamine in the present study, but lisdexamfetamine was not associated with sleep disturbances when used chronically (Adler et al., 2009a; Giblin and Strobel, 2011; Surman and Roth, 2011). Finally, we assessed the subjective effects of the substances in healthy subjects while abuse liability studies are typically conducted in substance-experienced subjects.

We are unaware of published direct comparisons of the pharmacokinetics of lisdexamfetamine and D-amphetamine. The present pharmacokinetic data for lisdexamfetamine and D-amphetamine are consistent with previous investigations of either formulation alone (Brown et al., 1979; Brauer et al., 1996; Krishnan et al., 2008; Ermer et al., 2010; Steer et al., 2012; Comiran et al., 2016; Adler et al., 2017). The present study showed that plasma amphetamine concentrations remained high after both lisdexamfetamine and D-amphetamine administration, with similarly long plasma elimination half-lives, consistent with previous studies (Angrist et al., 1987; Brauer et al., 1996; Krishnan et al., 2008). However, the present study illustrates that acute tolerance develops to the subjective drug effects, which were similar for both formulations. This means that the subjective stimulant drug effect lasts only up to 8 h, but plasma concentrations of amphetamine remain high. Similar to the present study, previous studies reported the development of acute tolerance to the subjective effects of D-amphetamine in healthy volunteers (Angrist et al., 1987; Brauer et al., 1996). In contrast to the present study, no tolerance to the subjective effects of methylphenidate was observed (Hysek et al., 2014). Another amphetamine derivative and serotonin and norepinephrine releaser, 3,4-methylenedioxymethamphetamine (MDMA), also presented marked acute pharmacological tolerance to both subjective and cardiovascular effects (Hysek et al., 2012, 2014). Tolerance was also observed after repeated daily oral administration of 10 mg methamphetamine (Kirkpatrick et al., 2012). D-amphetamine and methamphetamine act as indirect dopamine and norepinephrine agonists and release these catecholamines via their respective monoamine transporters (Simmler et al., 2013). In contrast, methylphenidate acts only as an inhibitor of the dopamine and norepinephrine transporter, without inducing their release (Simmler et al., 2013, 2014). Thus, monoamine depletion through release could potentially explain the phenomenon of acute tolerance to the subjective effects of D-amphetamine, in contrast to pure uptake inhibition by methylphenidate. However, this assumption is speculative and needs further study.

In rats, counterclockwise hysteresis was observed between the plasma concentration of amphetamine and locomotor activity after administration of lisdexamfetamine, but no such hysteresis was observed after D-amphetamine administration (Rowley et al., 2012). Additionally, counterclockwise hysteresis was observed between dopamine concentrations in the striatum and locomotor activity after lisdexamfetamine administration, but clockwise hysteresis was observed after D-amphetamine administration (Rowley et al., 2012). Similarly, in non-human primates, there was a counter-clockwise hysteresis between the plasma concentrations of amphetamine and the cocaine-like discriminative stimulus effects after administration of lisdexamfetamine intramuscularly (Banks et al., 2015). The differences between lisdexamfetamine and D-amphetamine in rats were considered to explain lower drug liking for lisdexamfetamine compared with D-amphetamine in humans (Rowley et al., 2012). However, in contrast to these preclinical data using parenteral drug administration, no differences were found in the hysteresis curves between lisdexamfetamine and D-amphetamine in the present study using oral drug administration, further supporting the similarity of the two substances when used orally in humans.

## Conclusion

The single oral dose pharmacokinetics and pharmacodynamics of lisdexamfetamine were similar to immediate-release D-amphetamine, although lisdexamfetamine had a longer lag time for the increase in plasma amphetamine concentration and subjective response. The risk of oral misuse of lisdexamfetamine is likely similar to D-amphetamine at least at relatively high doses.

## Author Contributions

Each of the authors participated in this research by contributing to the conception and design of the study (PD, PS, AO, and ML), study management (PD, PS, PV, and FH) performance of laboratory work (PS) and statistical analysis and interpretation (PD, PV, PS, FH, AO, and ML).

## Conflict of Interest Statement

The authors declare that the research was conducted in the absence of any commercial or financial relationships that could be construed as a potential conflict of interest.

## Supplementary Material

**FIGURE S1 |** Individual plasma amphetamine concentration-time curves. D-amphetamine was orally administered at a dose of 40 mg, and lisdexamfetamine was administered at a dose of 100 mg at *t* = 0 in the same subjects. The data represent individual observed plasma amphetamine concentrations measured at different time points (•, D-amphetamine; □, lisdexamfetamine) and amphetamine concentrations predicted by the one-compartment pharmacokinetic model (black lines). Note the longer lag time in the lisdexamfetamine condition in most subjects but the otherwise similar curve shapes, including similarly steep rates of increasing amphetamine concentrations.

**FIGURE S2 |** Lisdexamfetamine and D-amphetamine similarly increased subjective effects on the Adjective Mood Rating Scale compared with placebo. The data are expressed as the mean ± SEM in 24 subjects.

**FIGURE S3 |** Amphetamine concentration-subjective effect plots (hysteresis curves). To assess the amphetamine exposure-subjective effect relationship, the changes in pharmacodynamic effect after lisdexamfetamine and D-amphetamine administration compared with placebo for each time point were plotted against the respective plasma concentrations of amphetamine (hysteresis plots). The subjective effects presented similar clockwise hysteresis after administration of lisdexamfetamine and D-amphetamine, indicating comparable acute pharmacological tolerance. Thus, the subjective effects decreased despite the continued high plasma concentration of amphetamine after administration of both drugs. The data are expressed as mean ± SEM. The time of sampling is noted next to each point. The drugs were administered at *t* = 0.

**FIGURE S4 |** The pupil diameter at rest in the dark or after a light stimulus changed similarly over time after administration of lisdexamfetamine and D-amphetamine compared with placebo. The data are expressed as the mean ± SEM in 24 subject.

**FIGURE S5 |** Amphetamine concentration-cardiovascular effect plots (hysteresis curves). To assess the amphetamine exposure-cardiovascular response relationship, the changes in vital signs after lisdexamfetamine and D-amphetamine administration compared with placebo for each time point were plotted against the respective plasma concentrations of amphetamine (hysteresis plots). The blood pressure response presented similar clockwise hysteresis after administration of lisdexamfetamine and D-amphetamine, indicating comparable acute pharmacological tolerance. The heart rate response presented similar counterclockwise hysteresis after administration of lisdexamfetamine and D-amphetamine, indicating a comparable lag and the absence of tolerance. The rate pressure product as a measure of the overall cardiovascular response (systolic blood pressure × heart rate) showed no tolerance in contrast to the subjective drug effects (**Supplementary Figure S3**). The data are expressed as mean ± SEM. The time of sampling is noted next to each point. The drugs were administered at *t* = 0.
