# Supplementary material for: Pharmacokinetics and Pharmacodynamics of Lisdexamfetamine Compared with D-Amphetamine in Healthy Subjects
Source: Front Pharmacol. 2017 Sep 7;8:617. doi: 10.3389/fphar.2017.00617 (PMC5594082; doi:10.3389/fphar.2017.00617)
Supplement: Supplementary file 1 [file Supplementary_Figure_S1.DOCX]

| 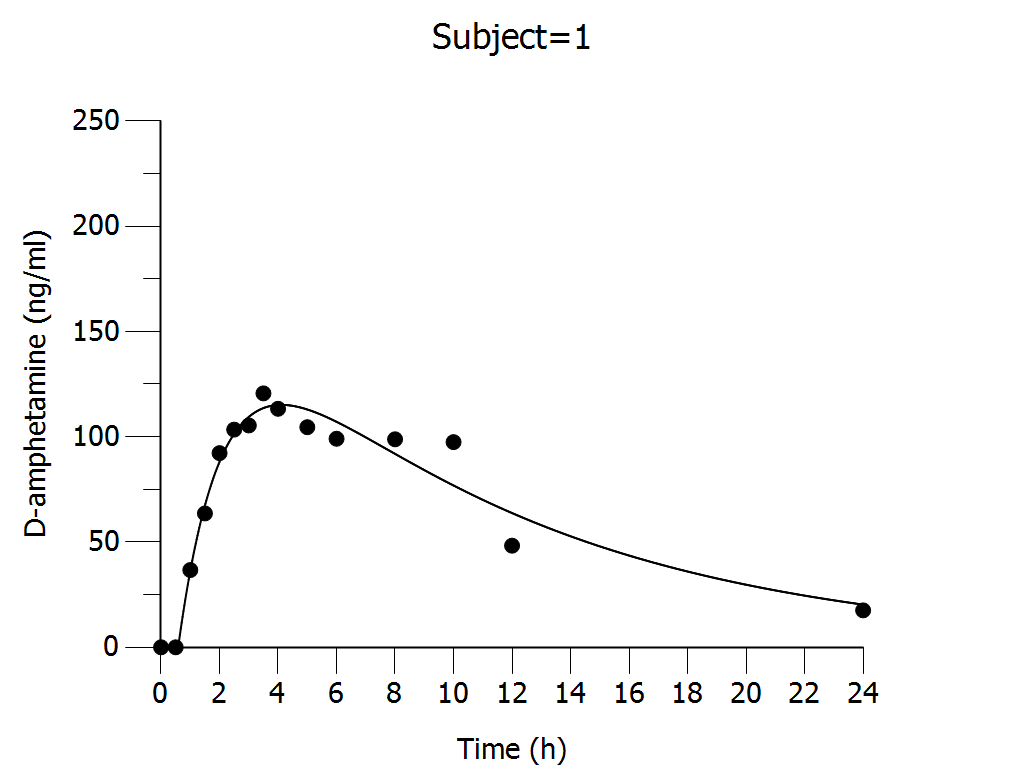 | 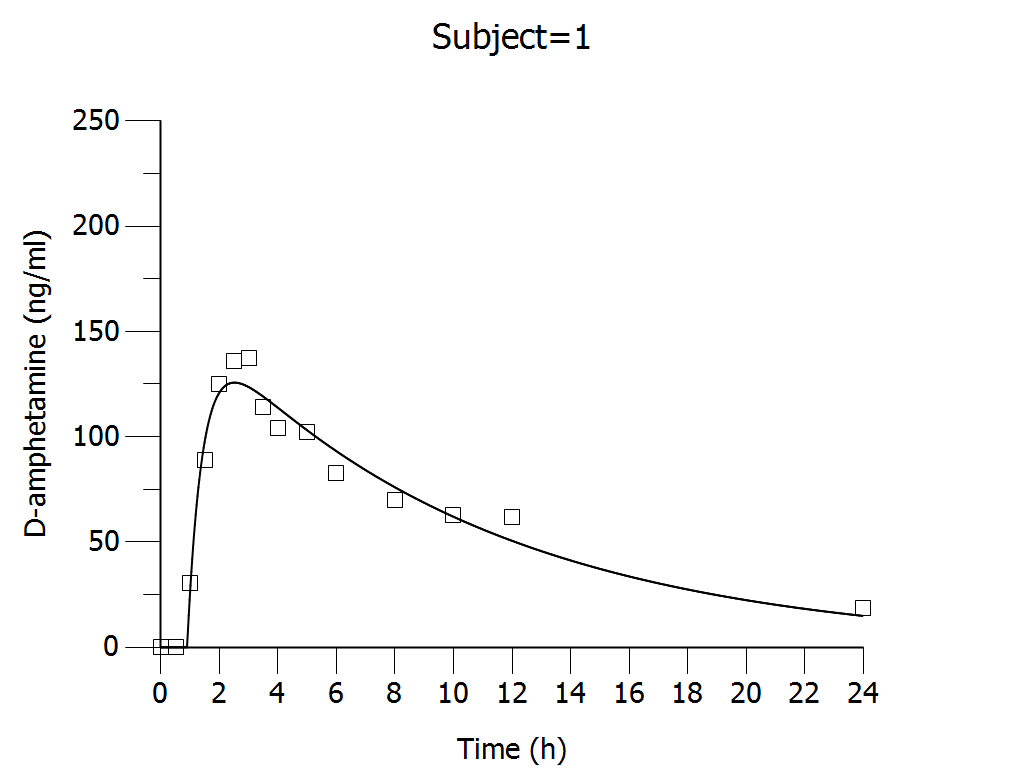 |
| --- | --- |
| 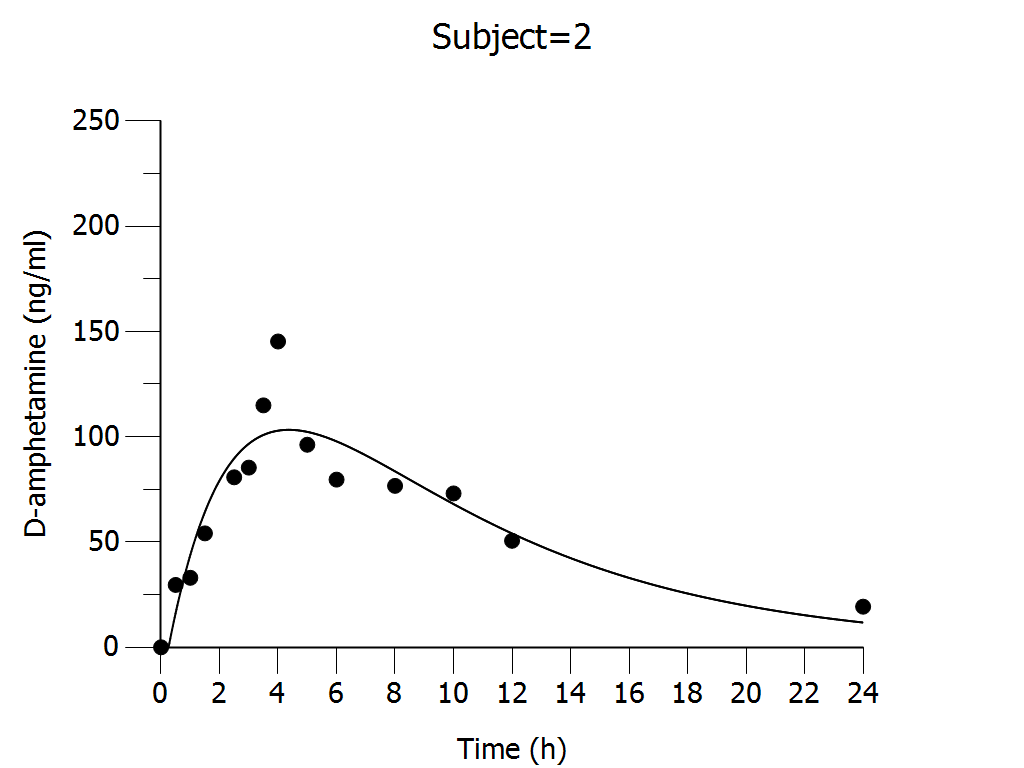 | 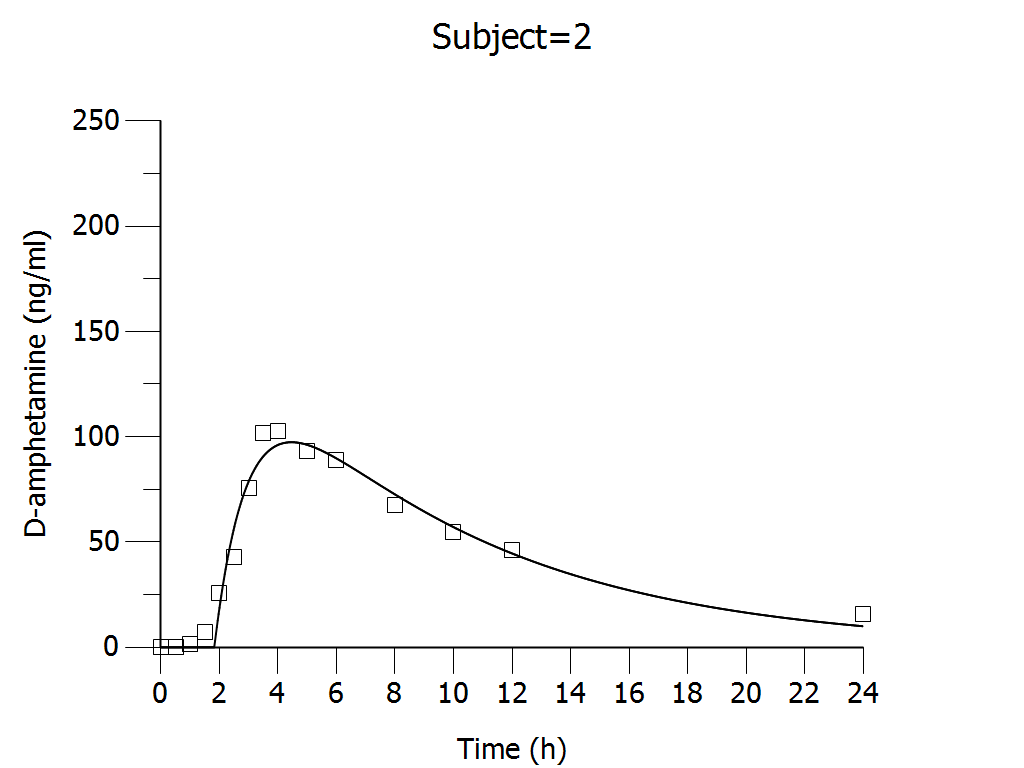 |
| 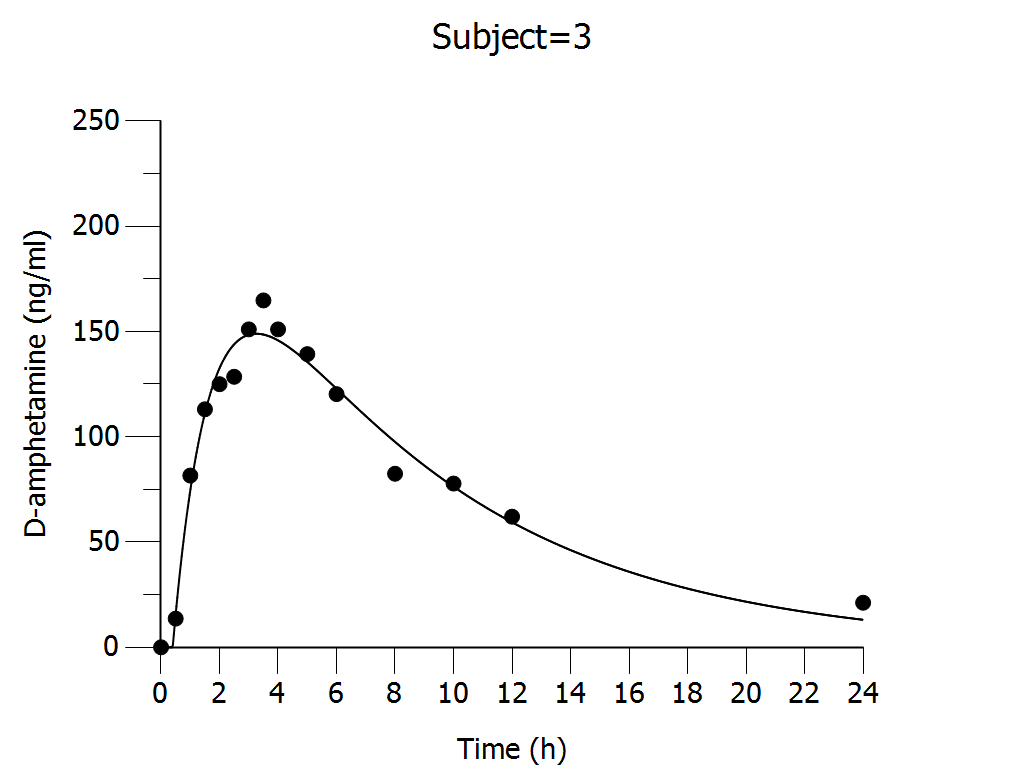 | 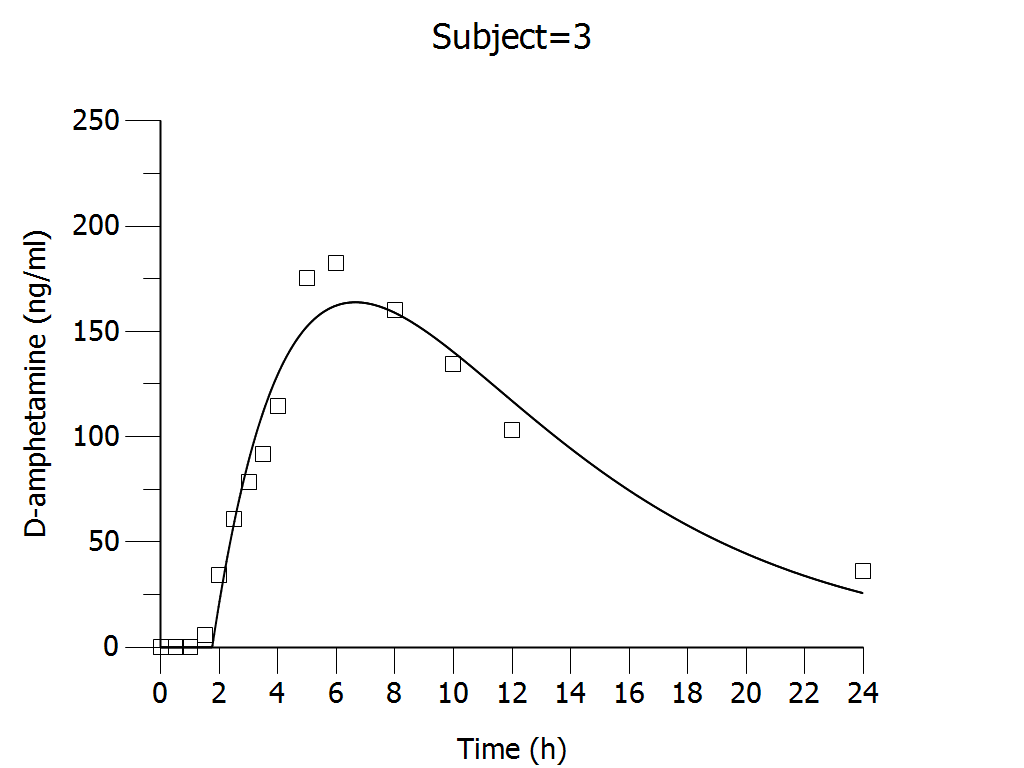 |
| 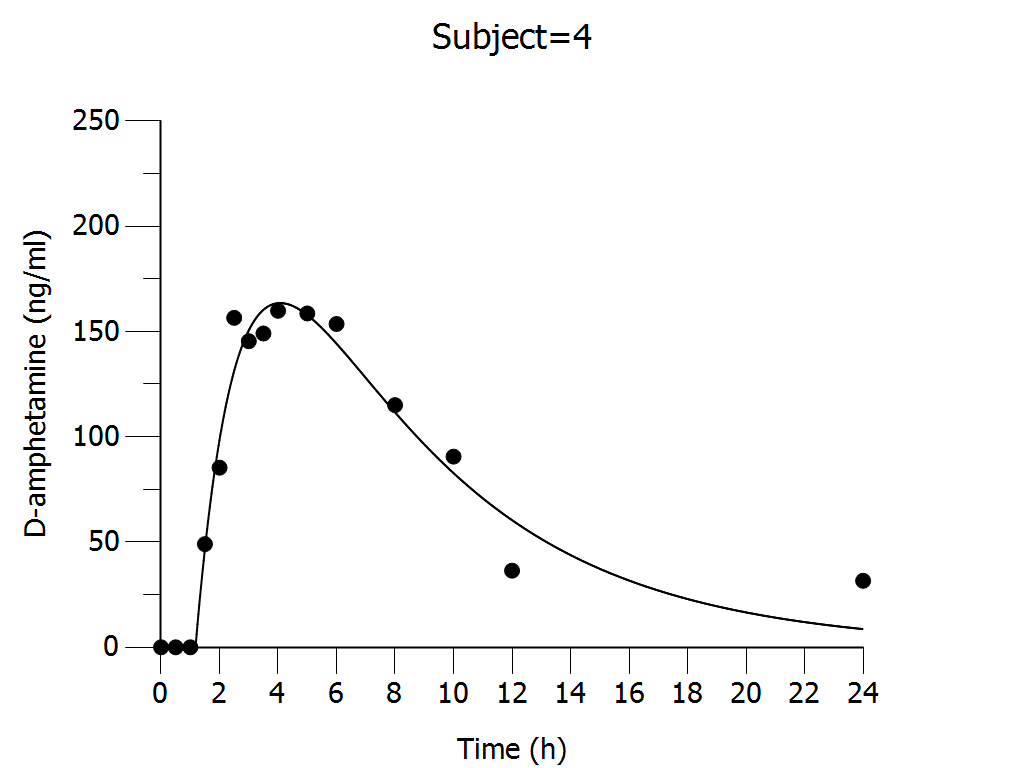 | 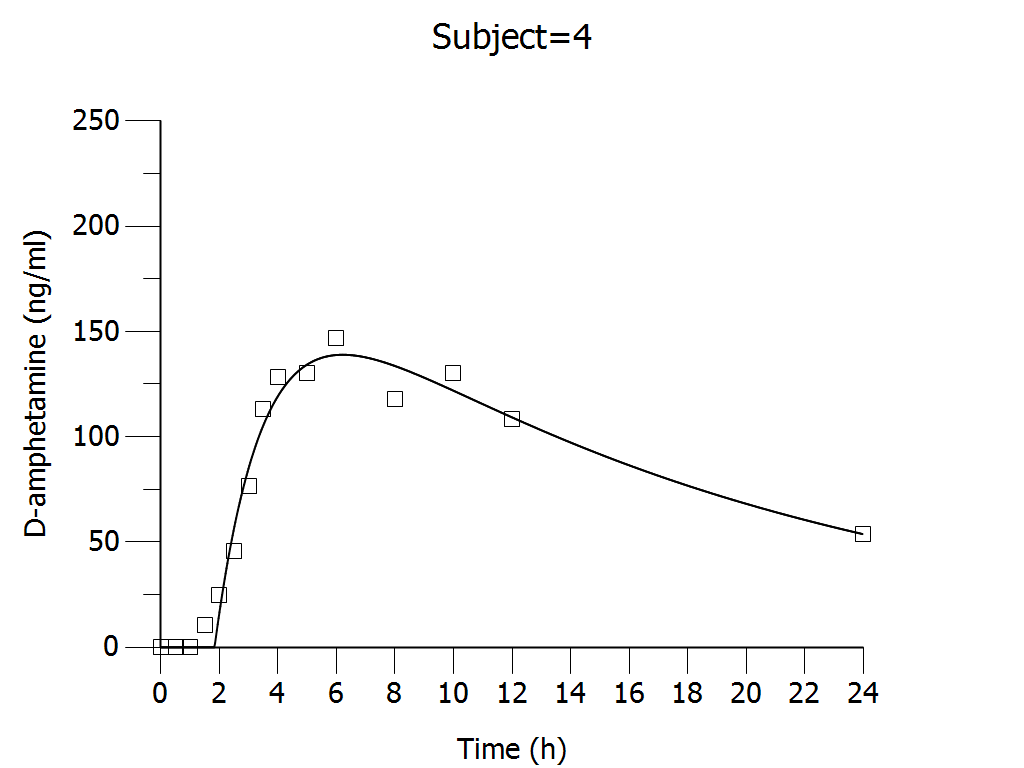 |
| 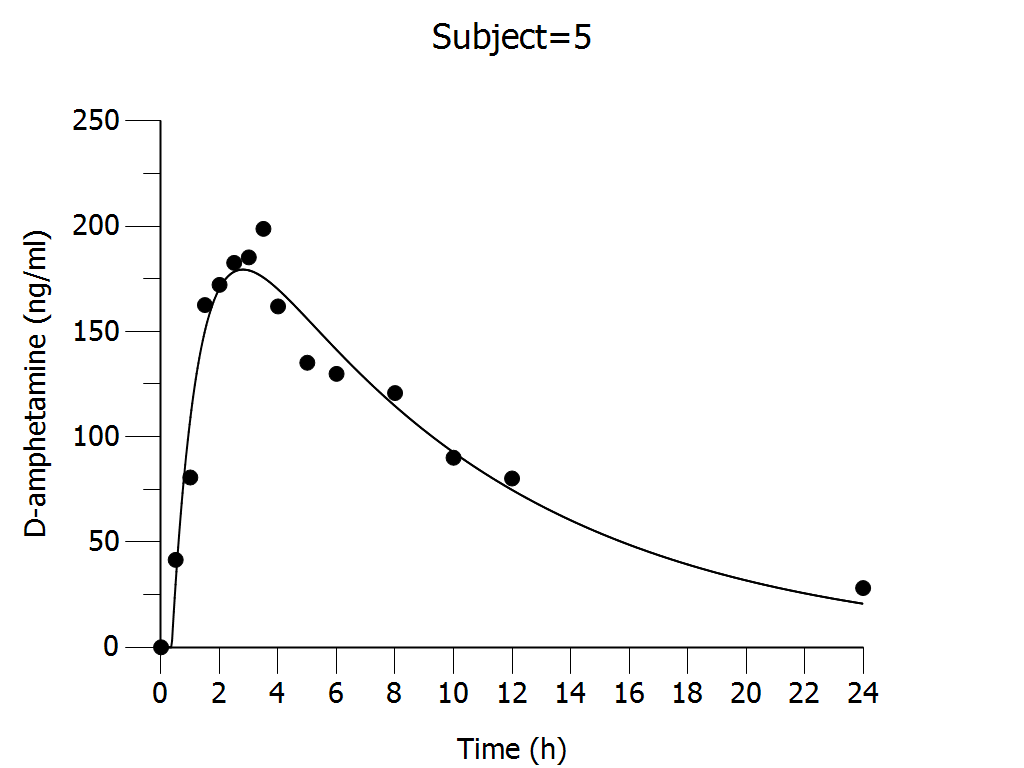 | 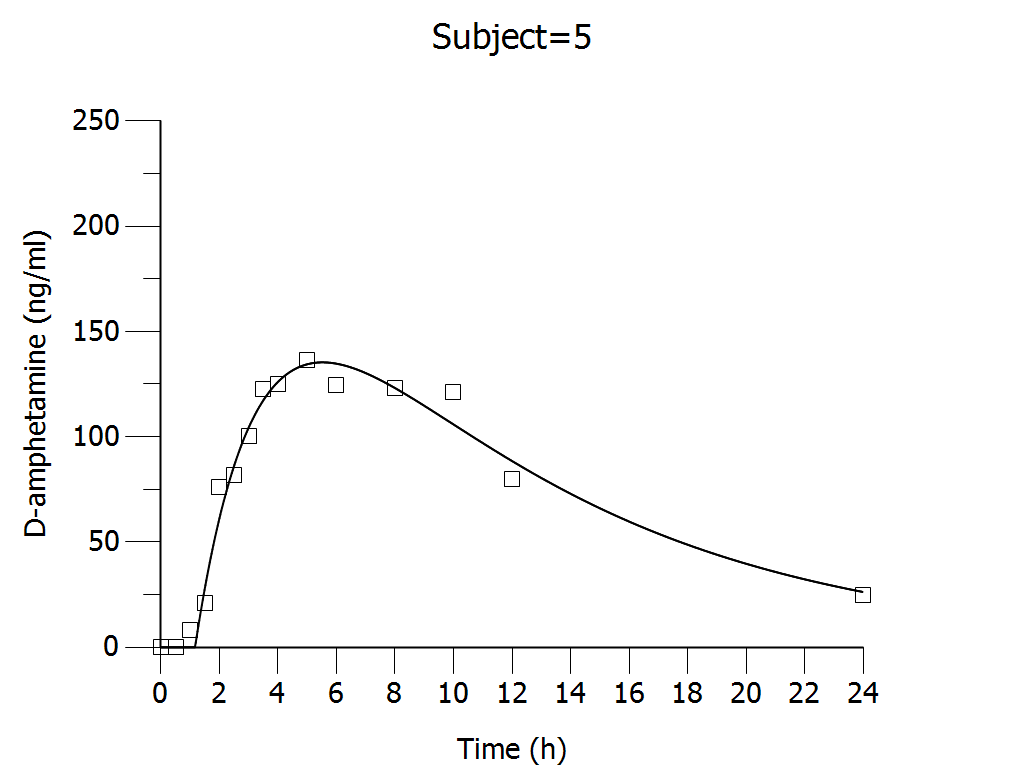 |
| 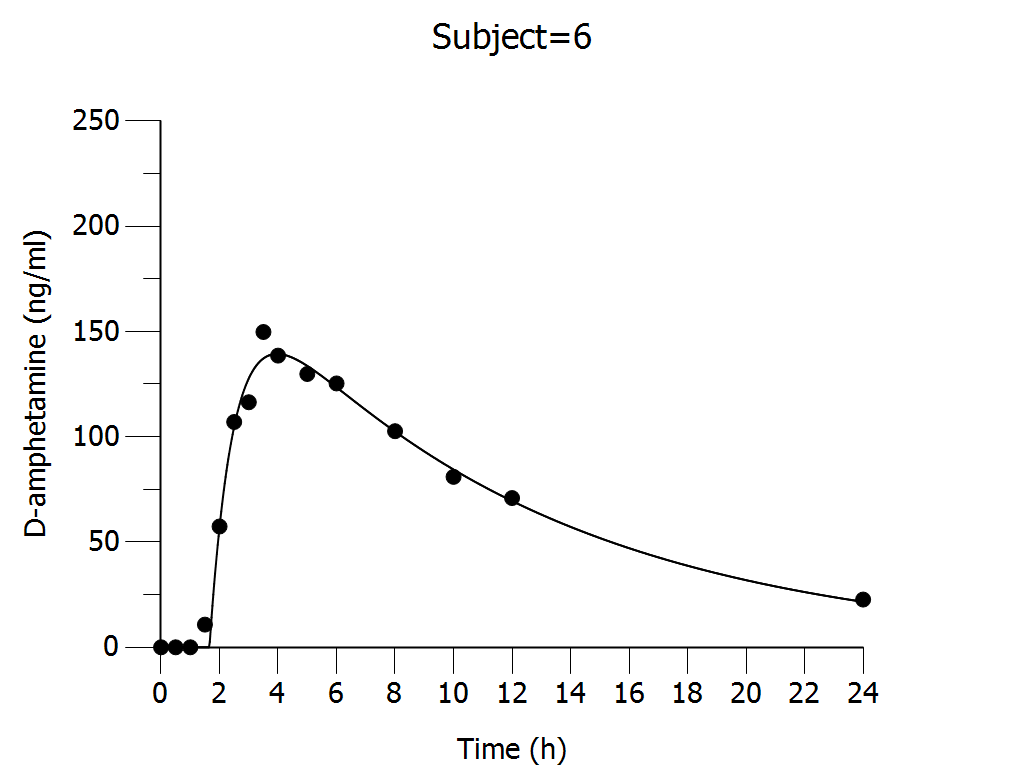 | 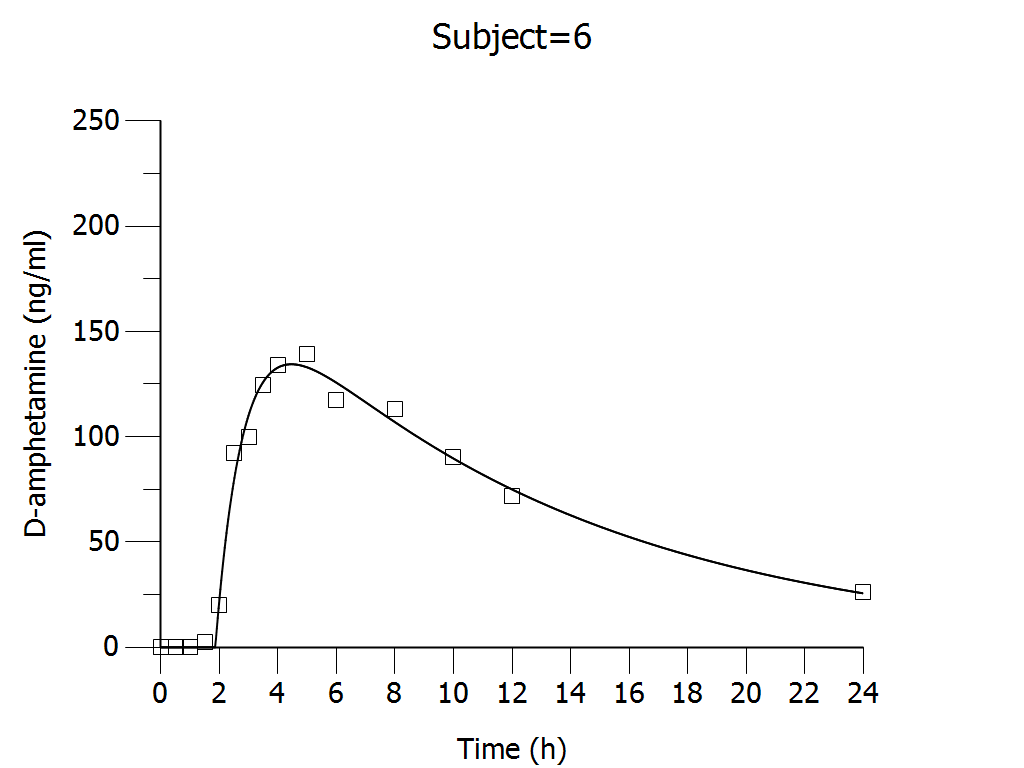 |
| 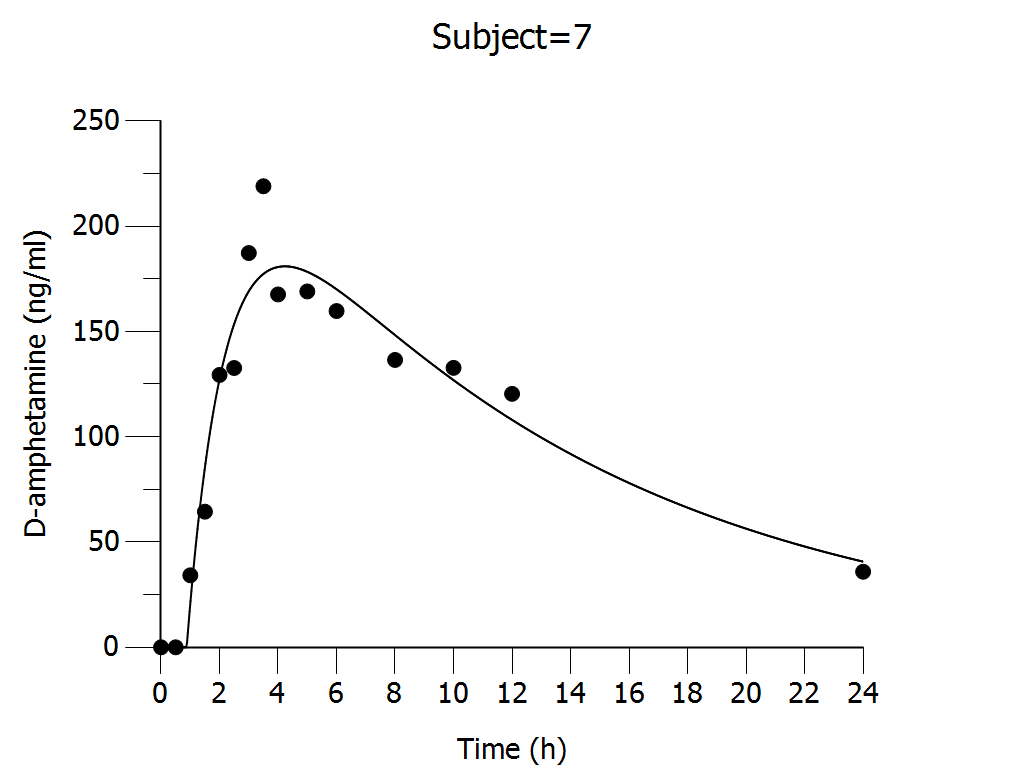 | 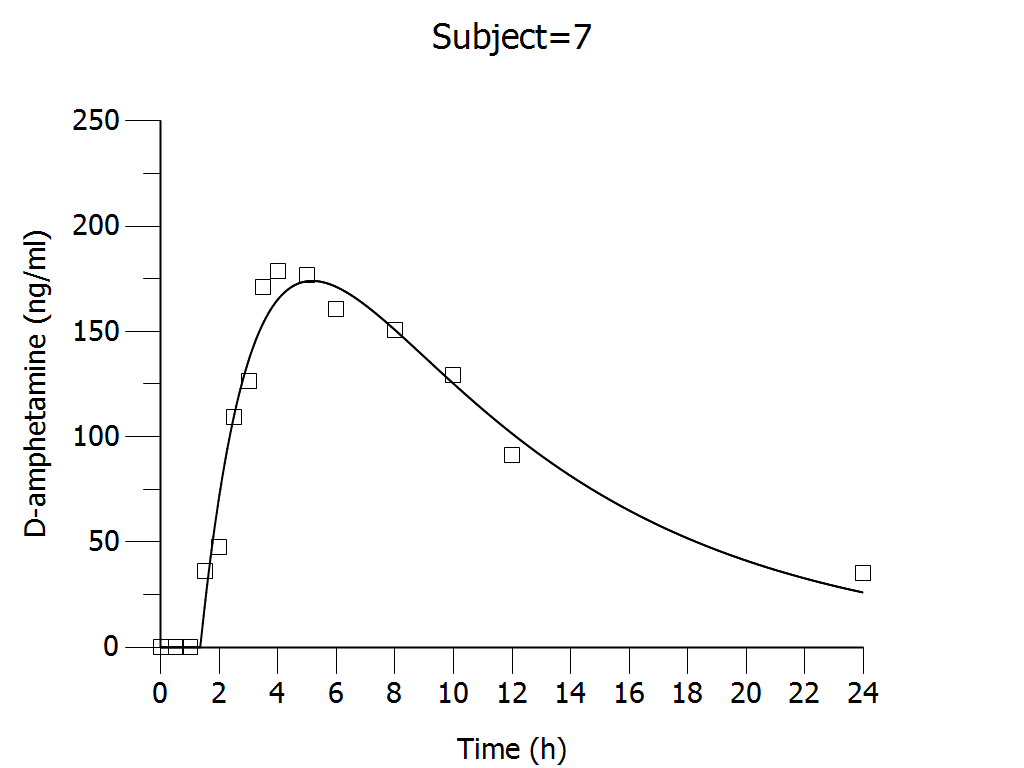 |
| 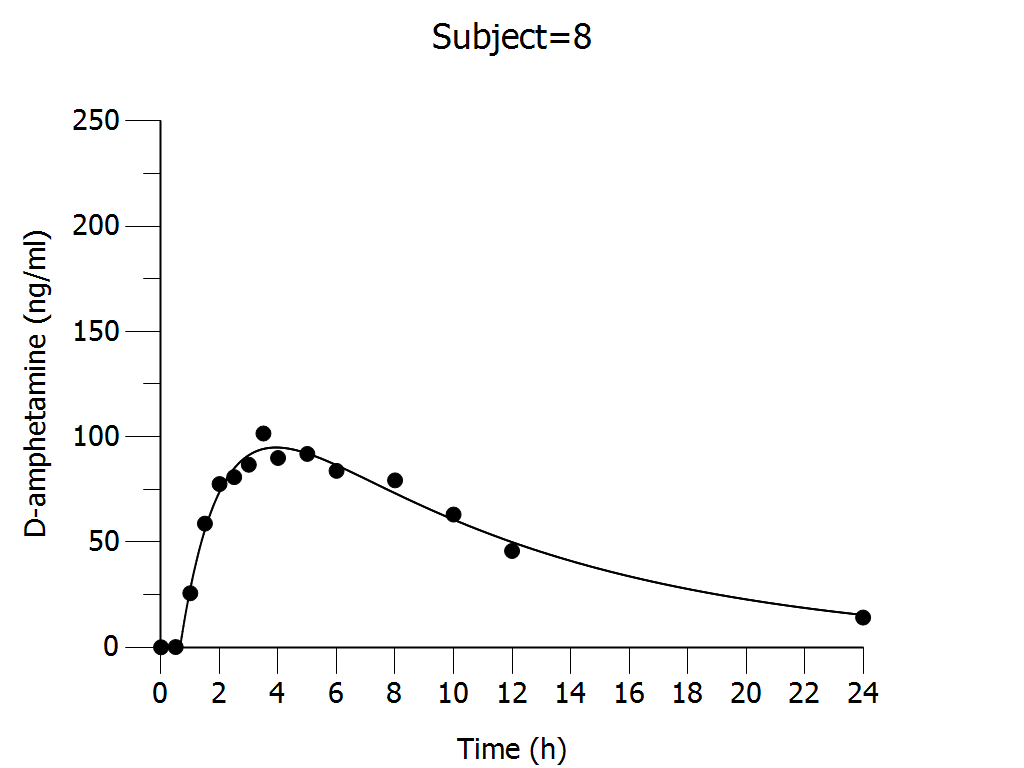 | 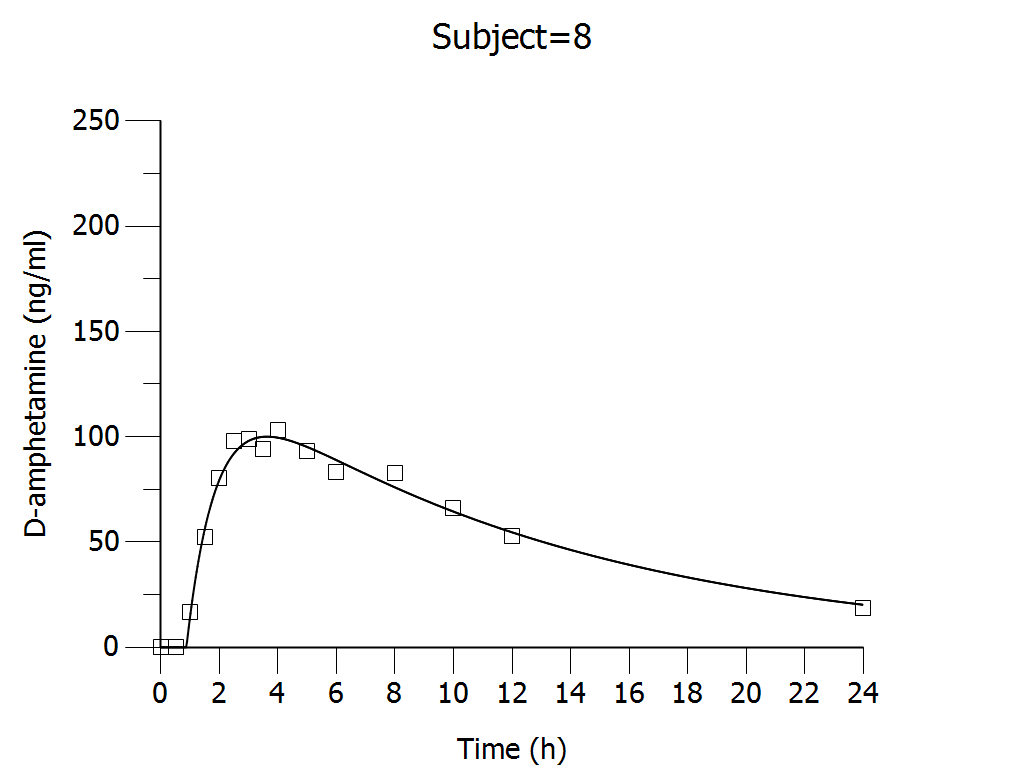 |
| 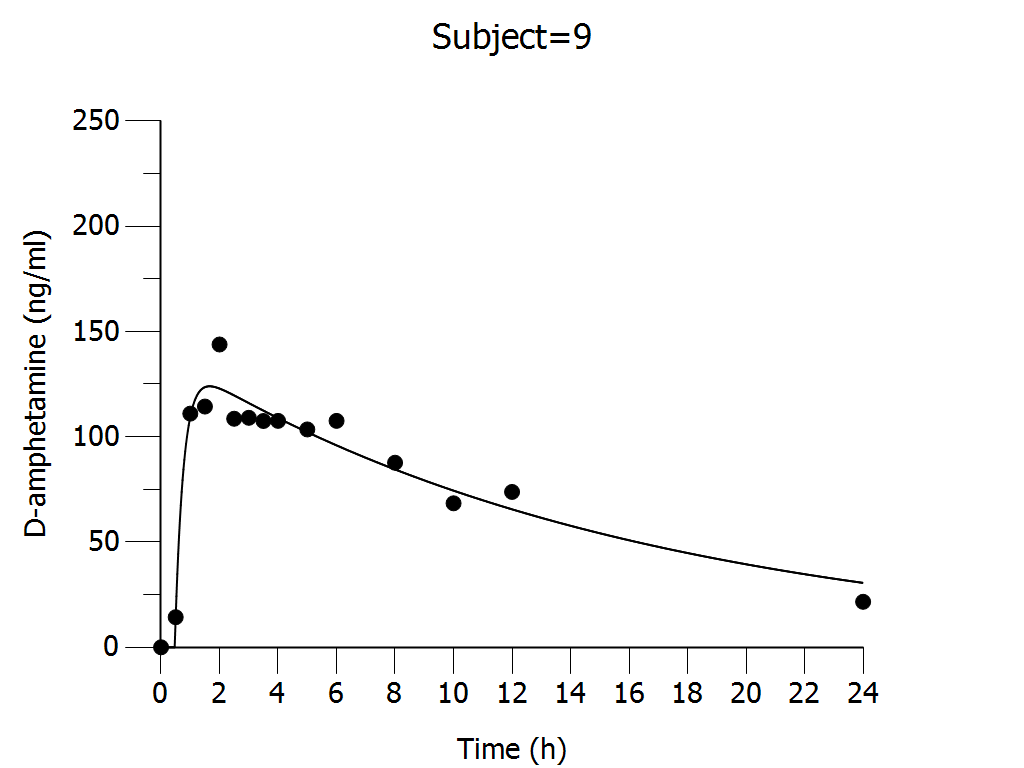 | 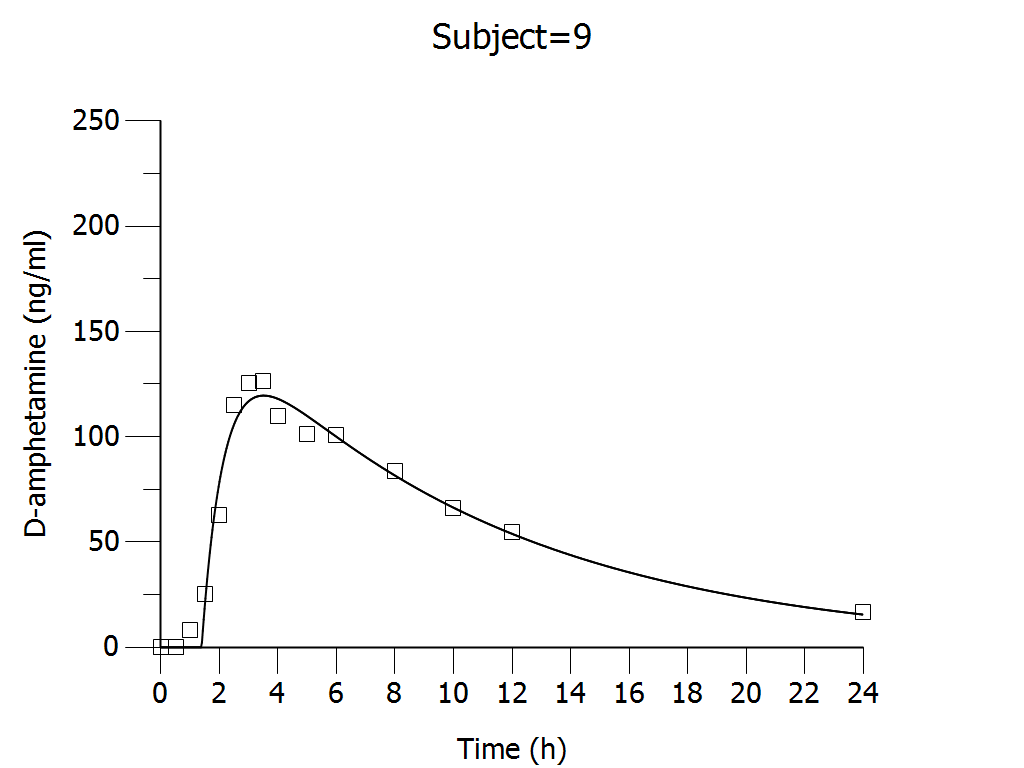 |
| 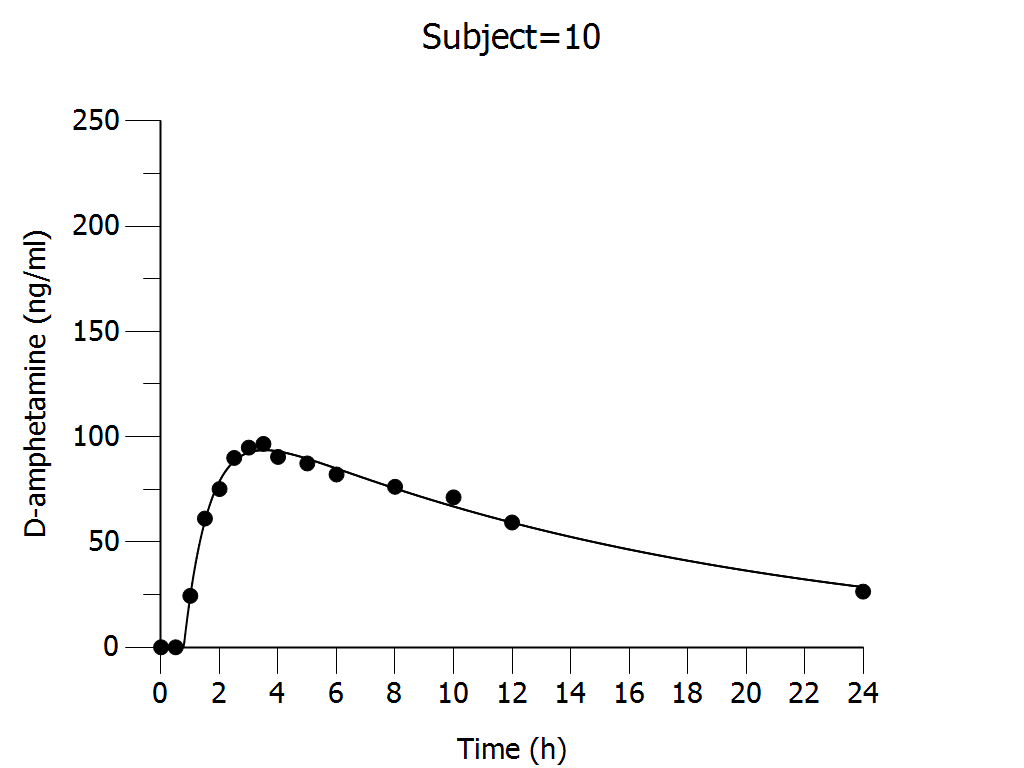 | 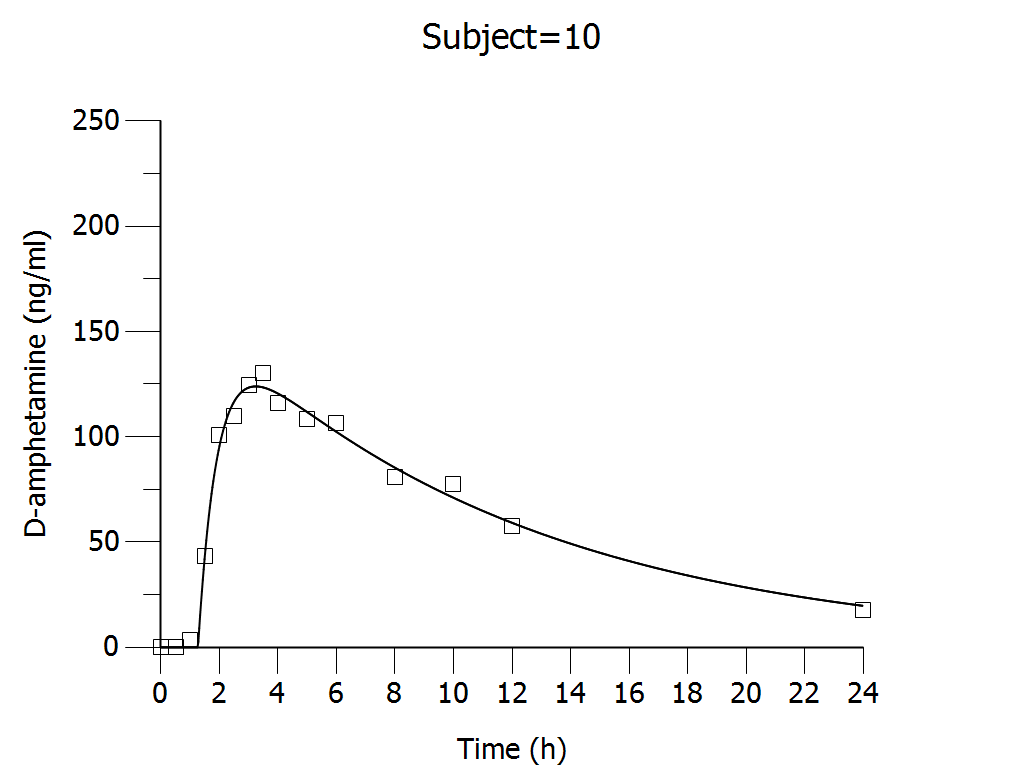 |
| 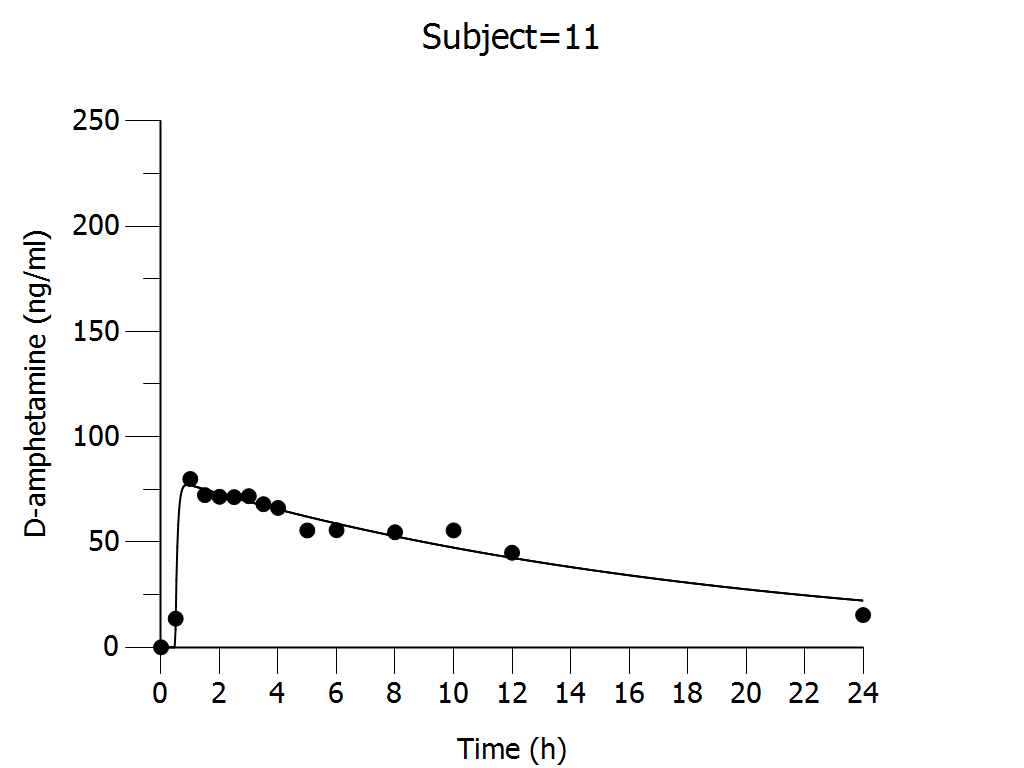 | 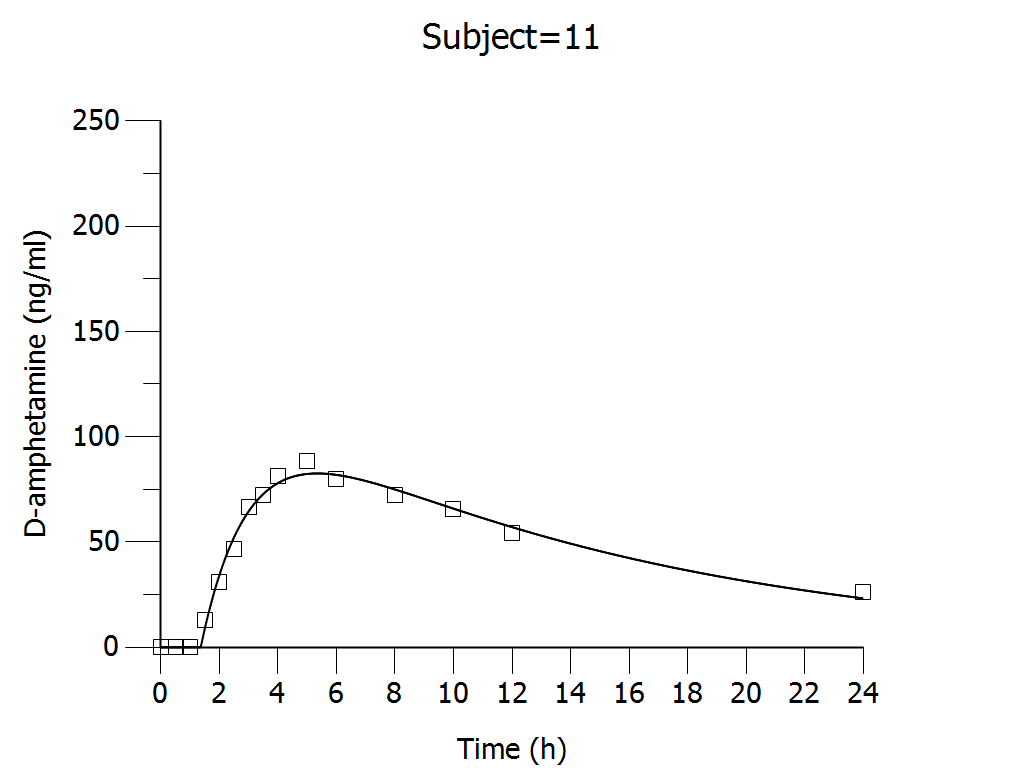 |
| 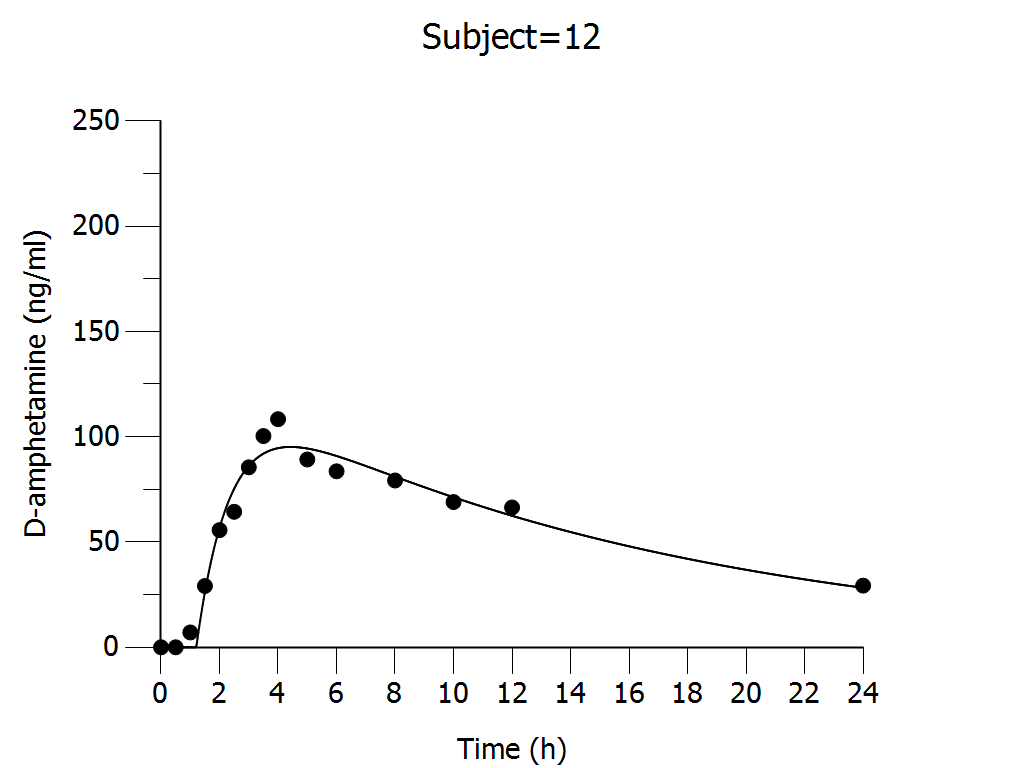 | 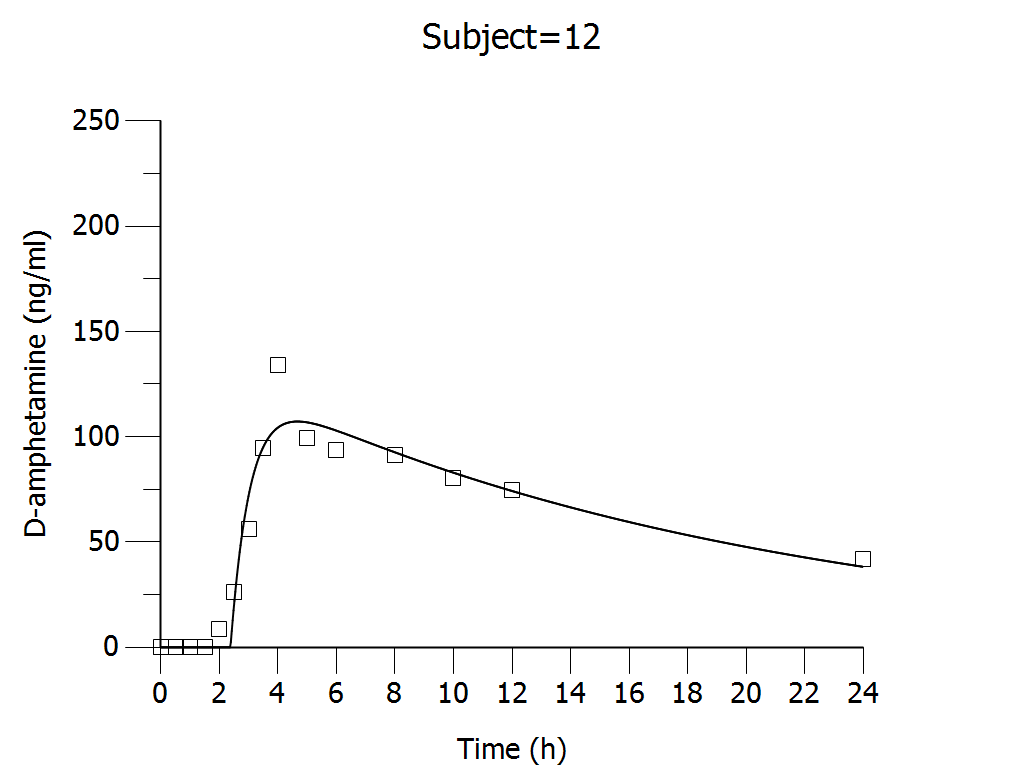 |
| 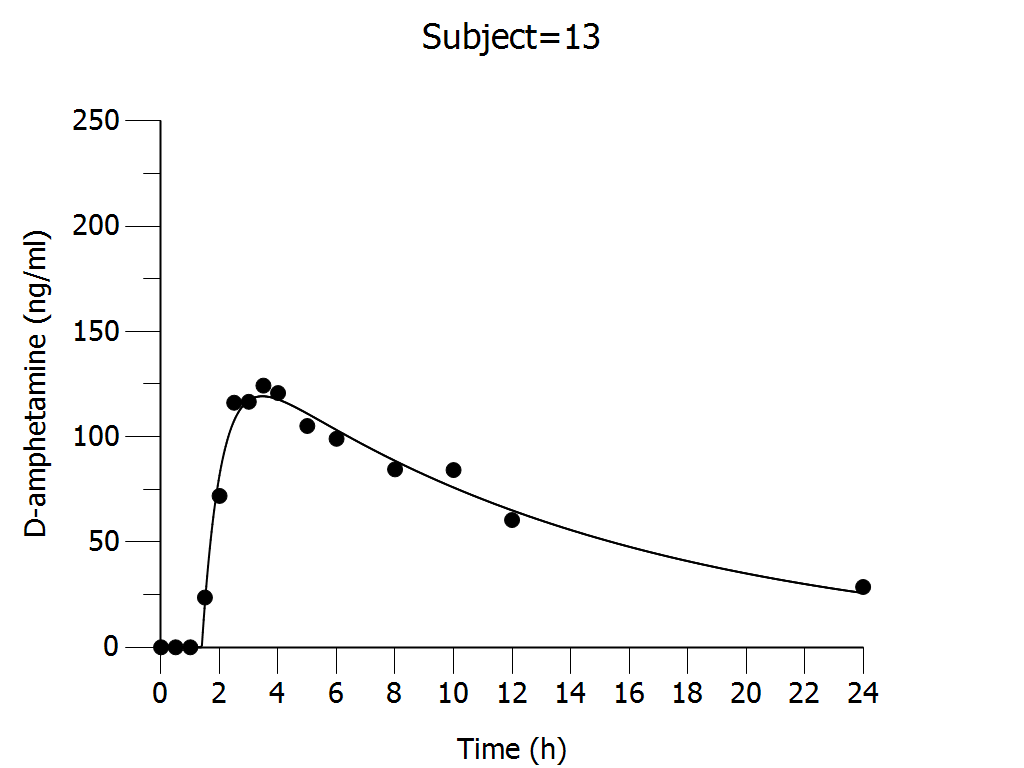 | 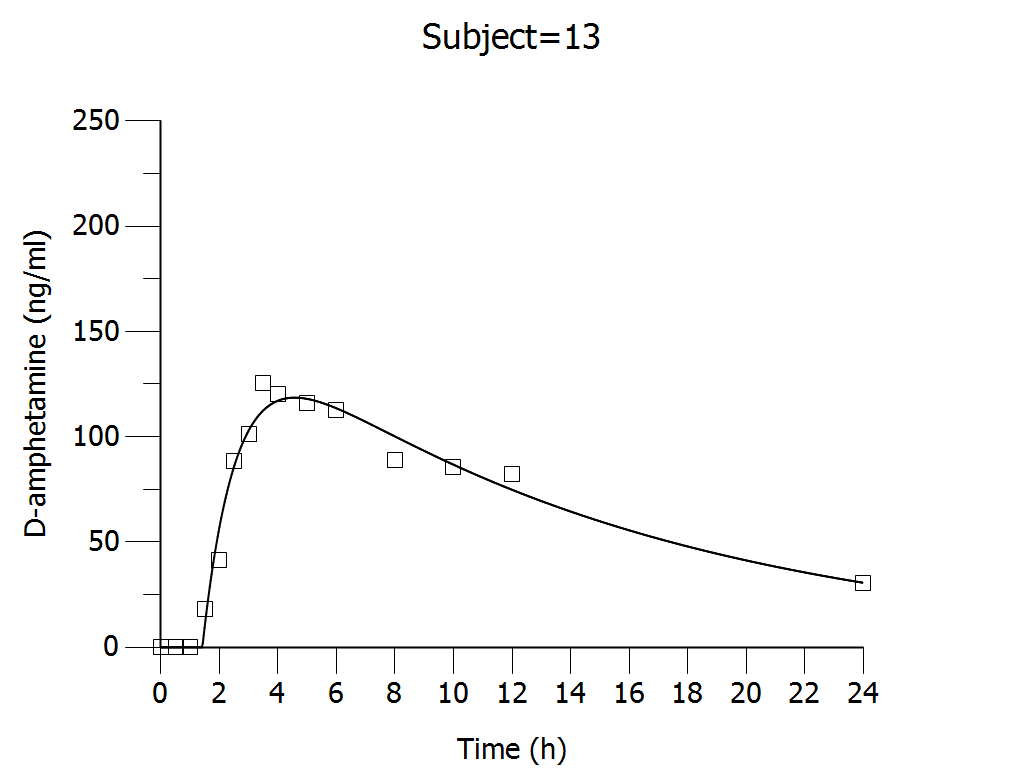 |
| 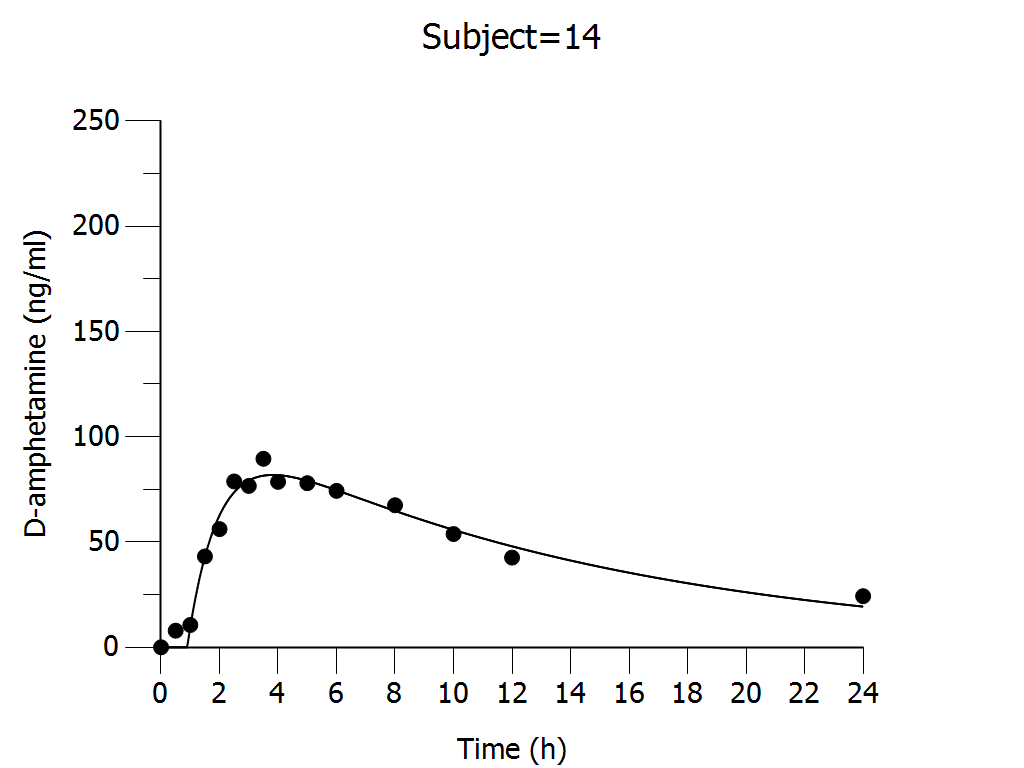 | 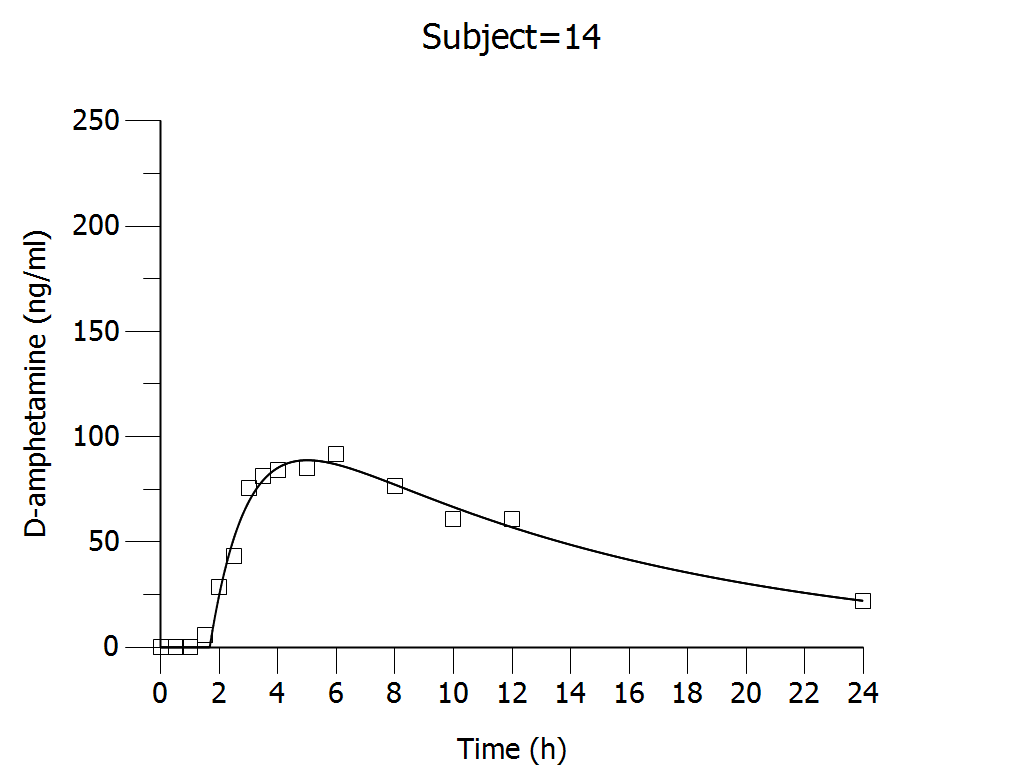 |
| 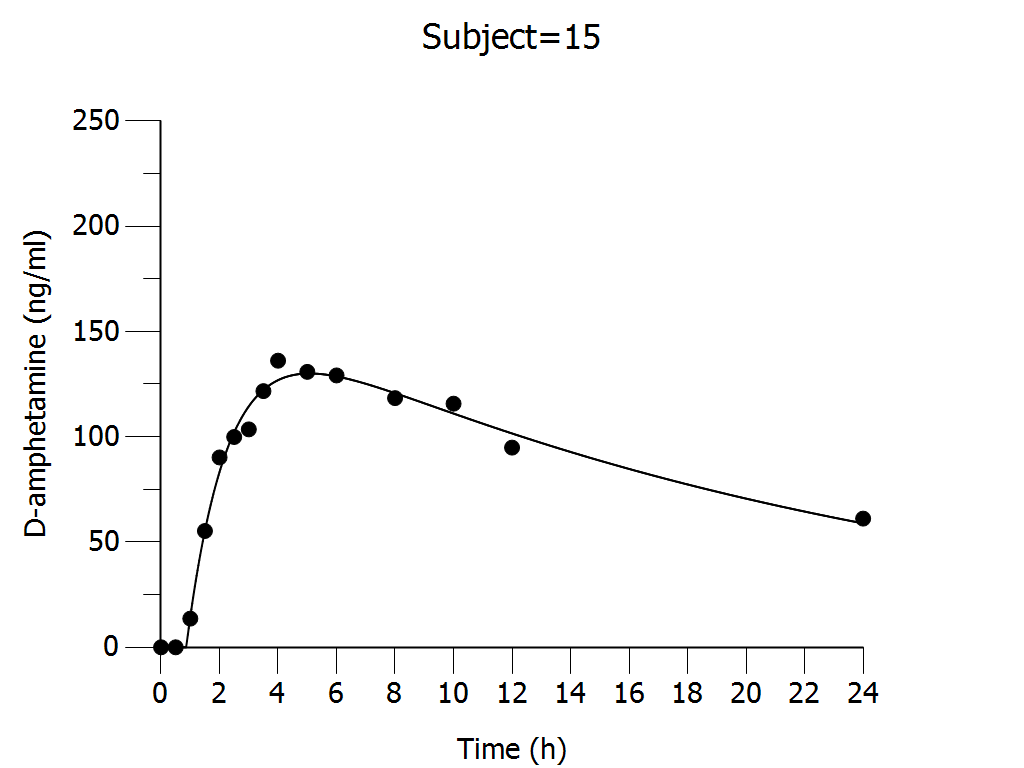 | 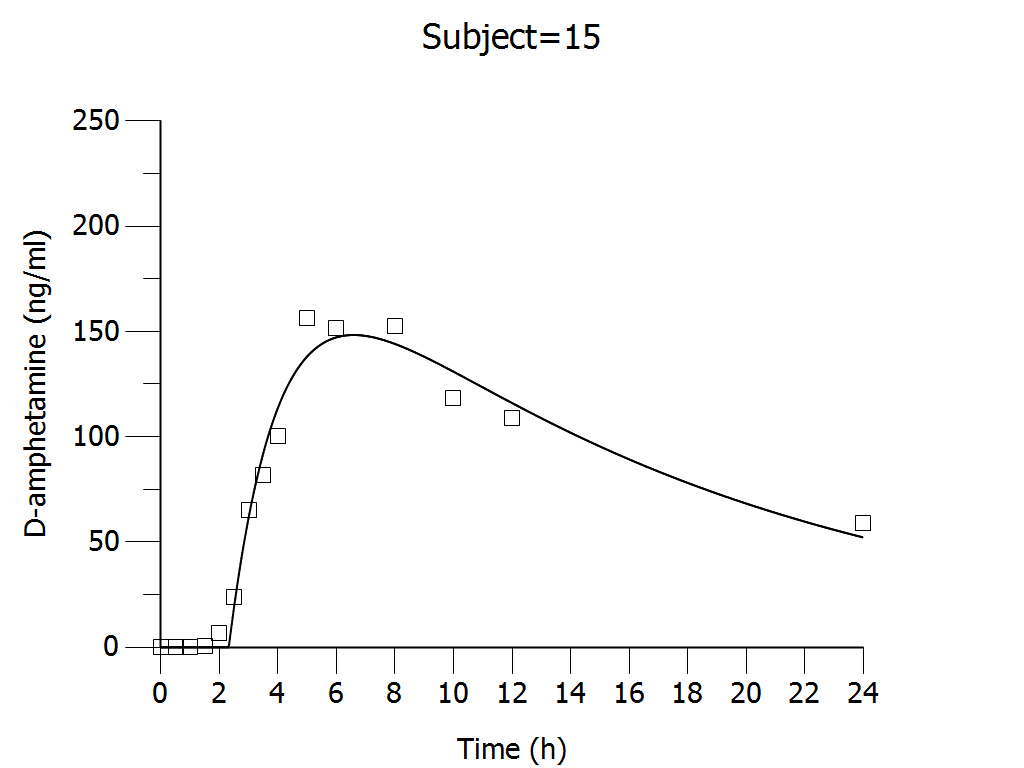 |
| 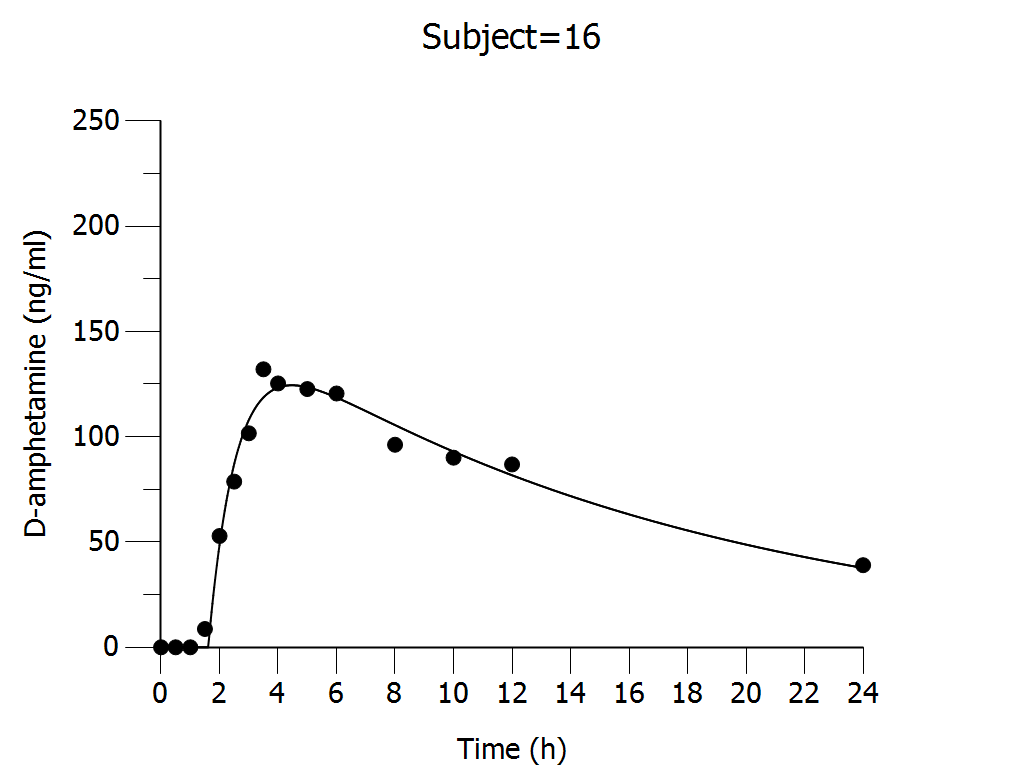 | 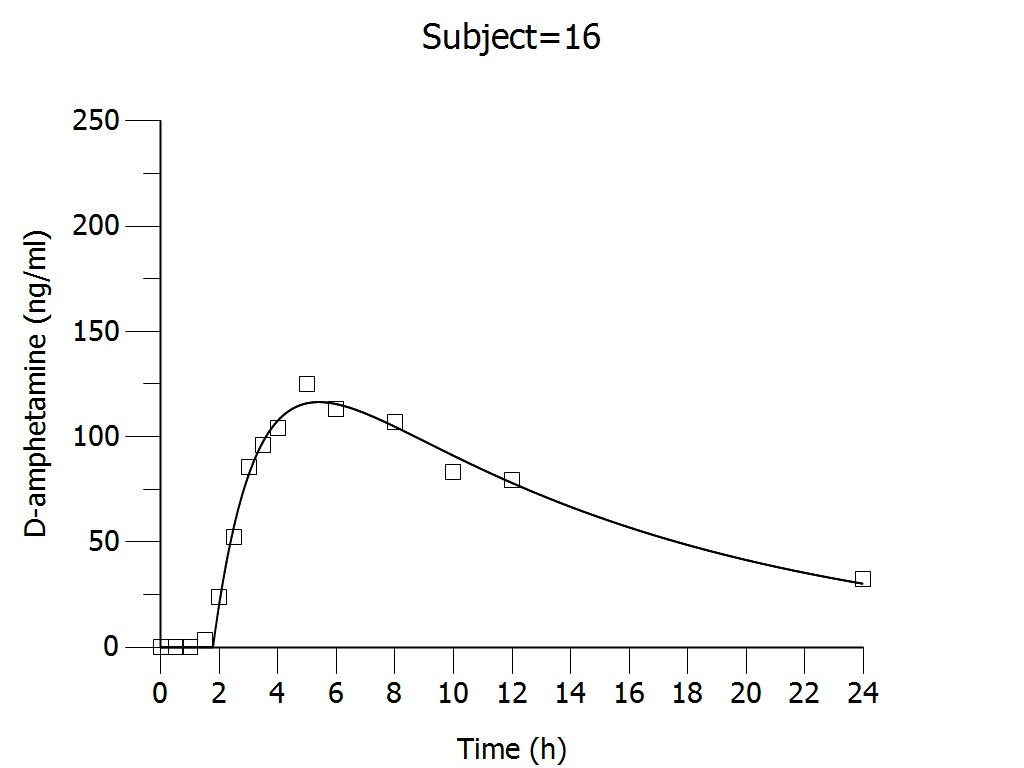 |
| 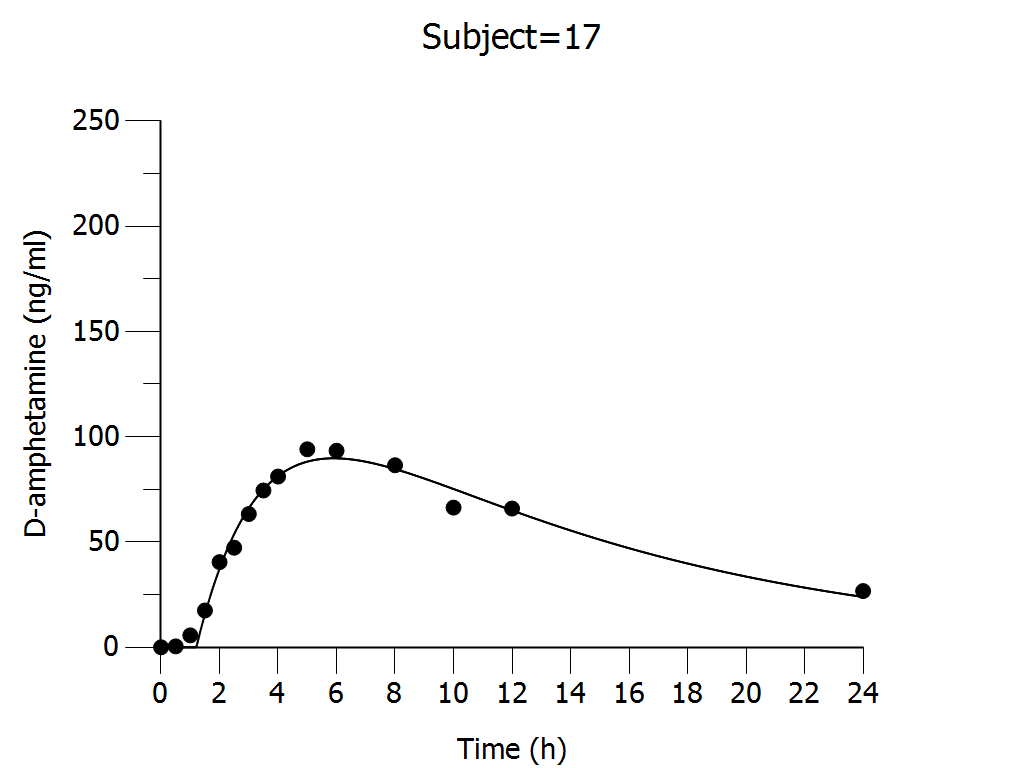 | 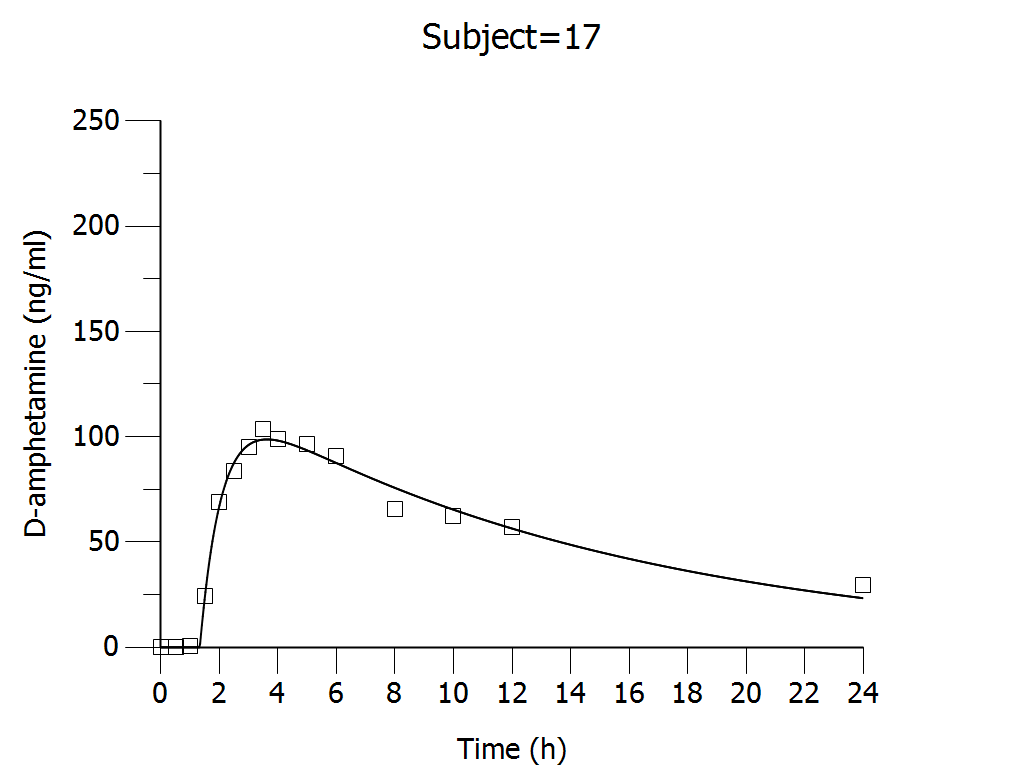 |
| 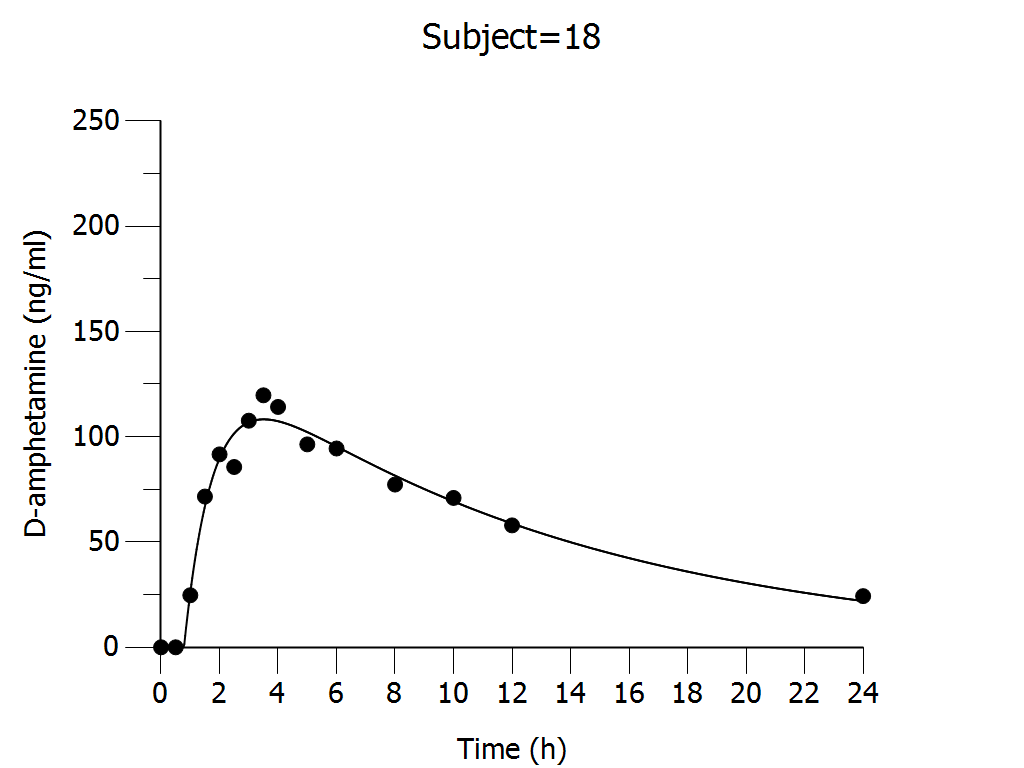 | 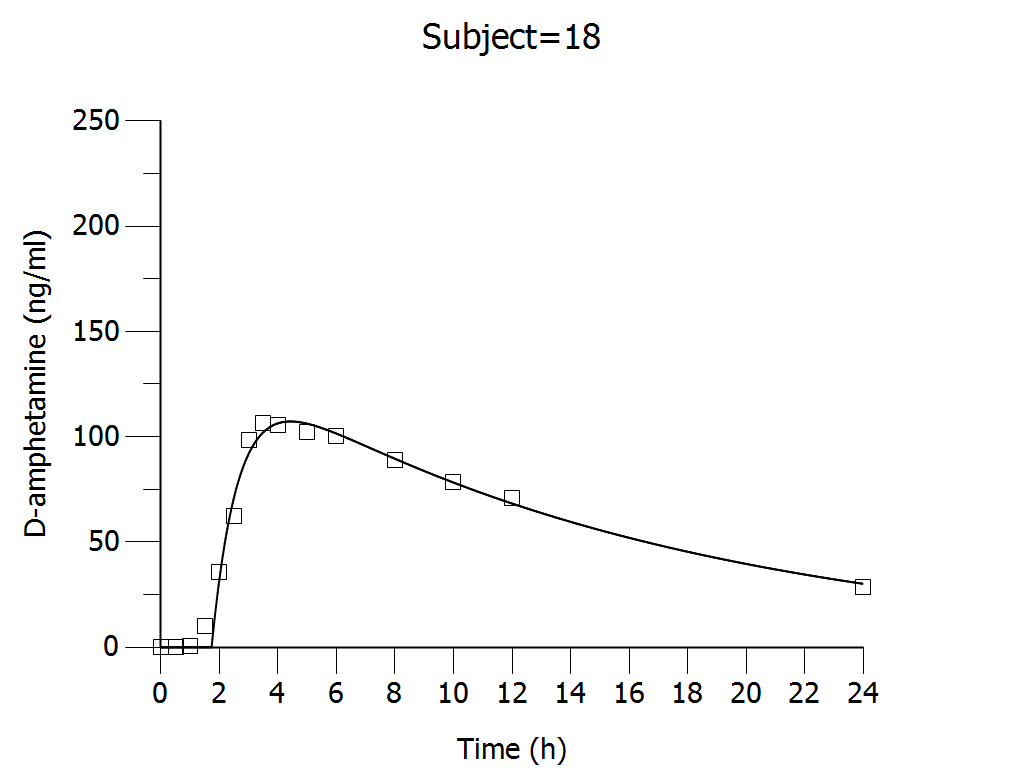 |
| 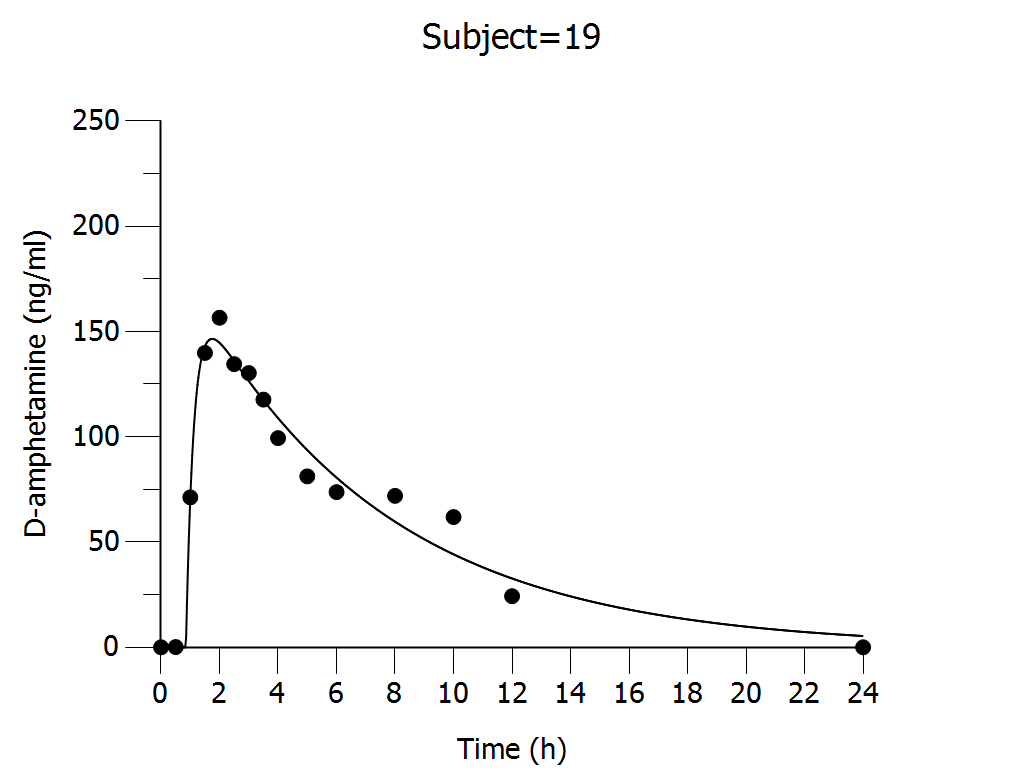 | 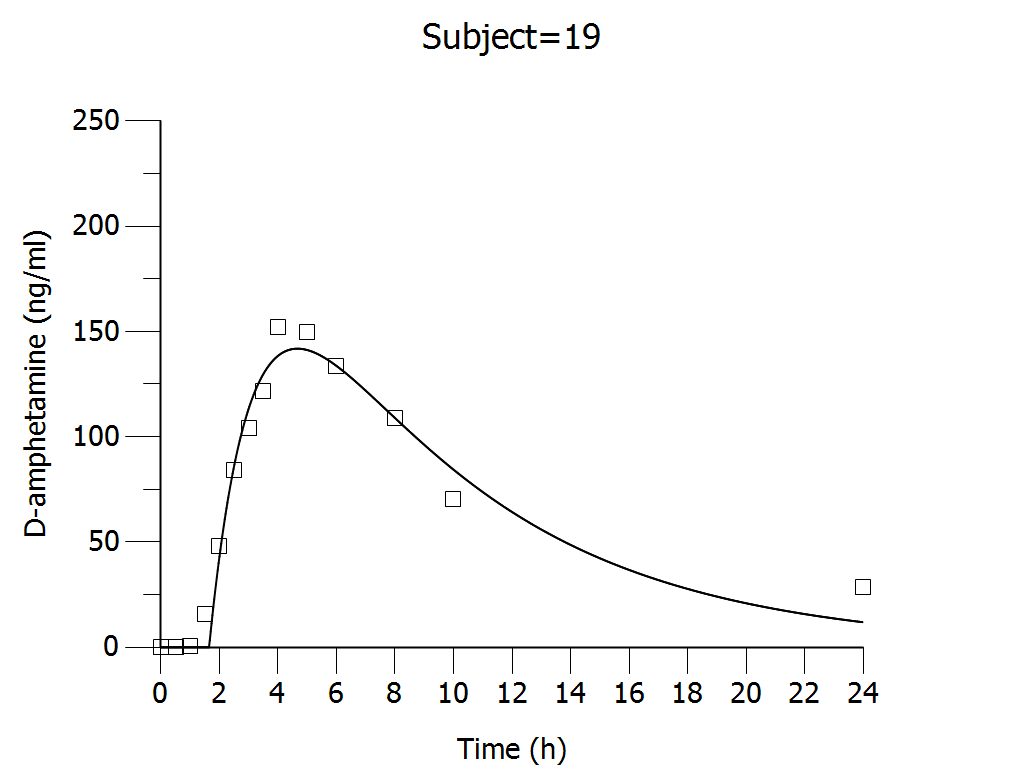 |
| 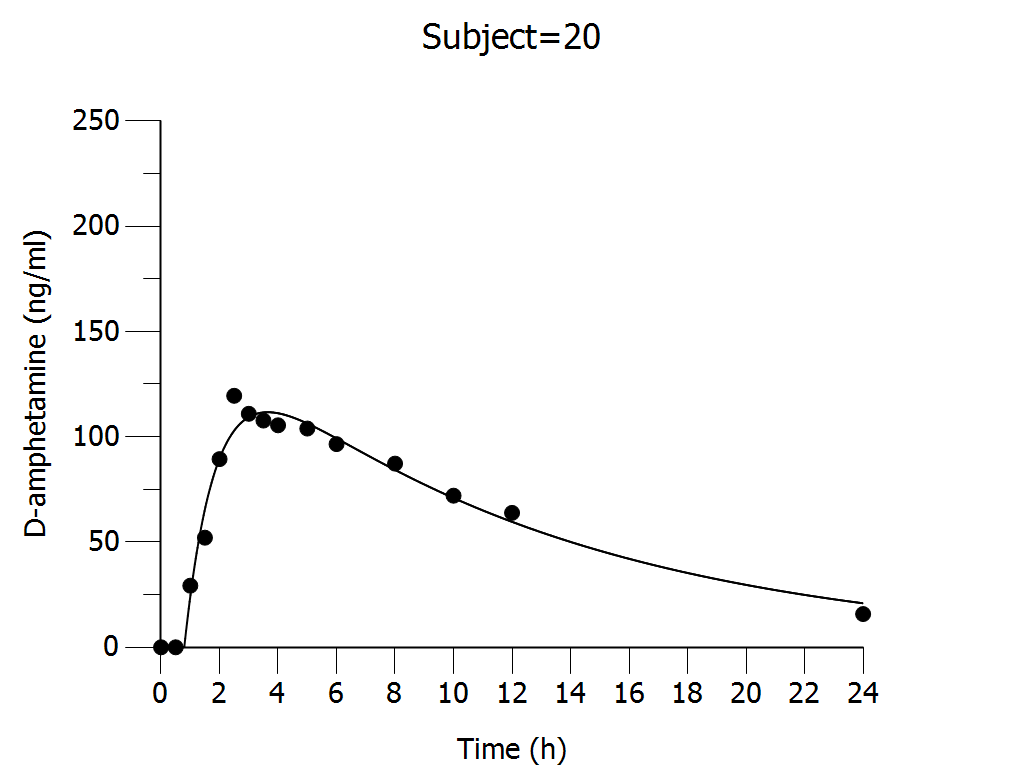 | 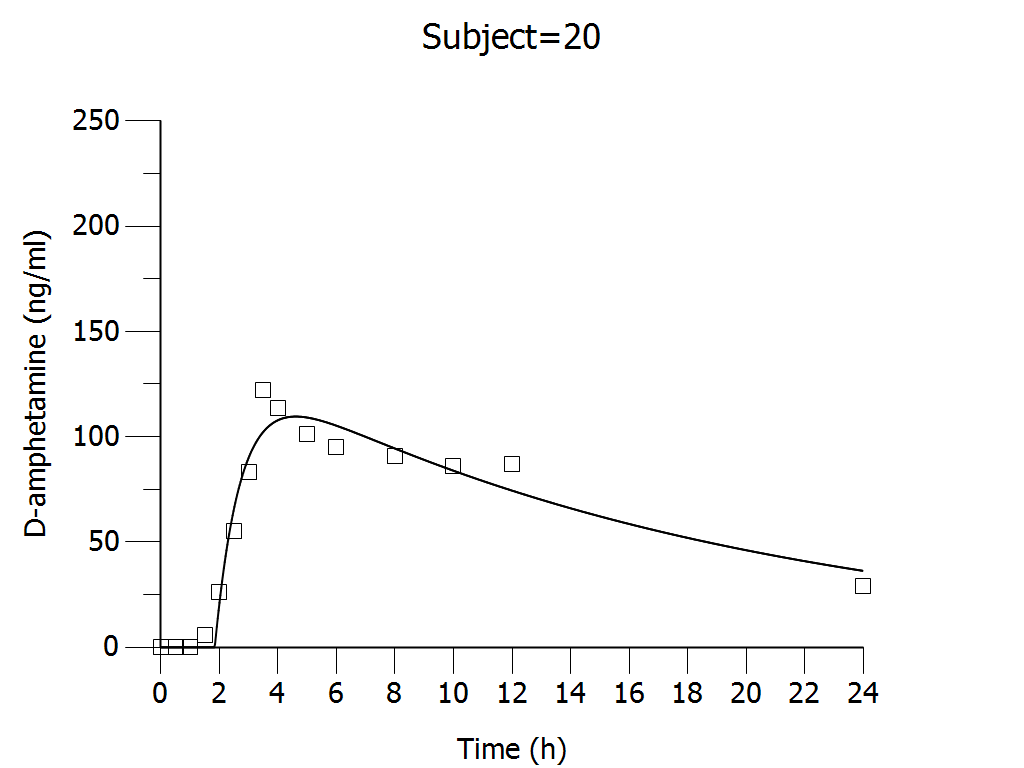 |
| 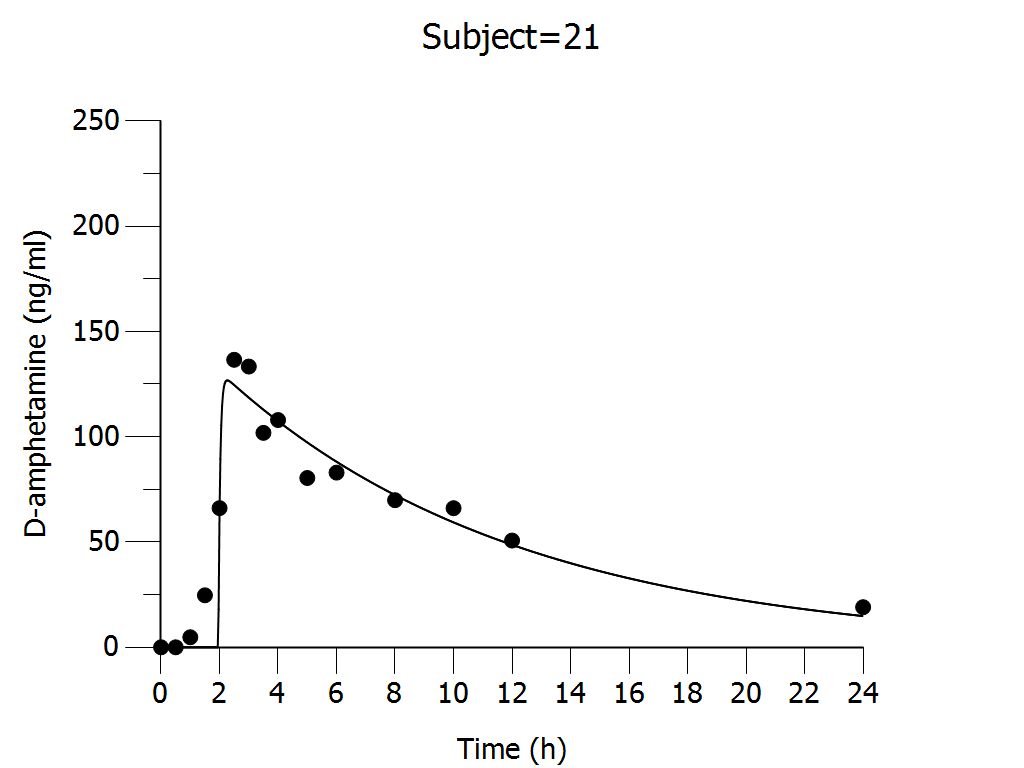 | 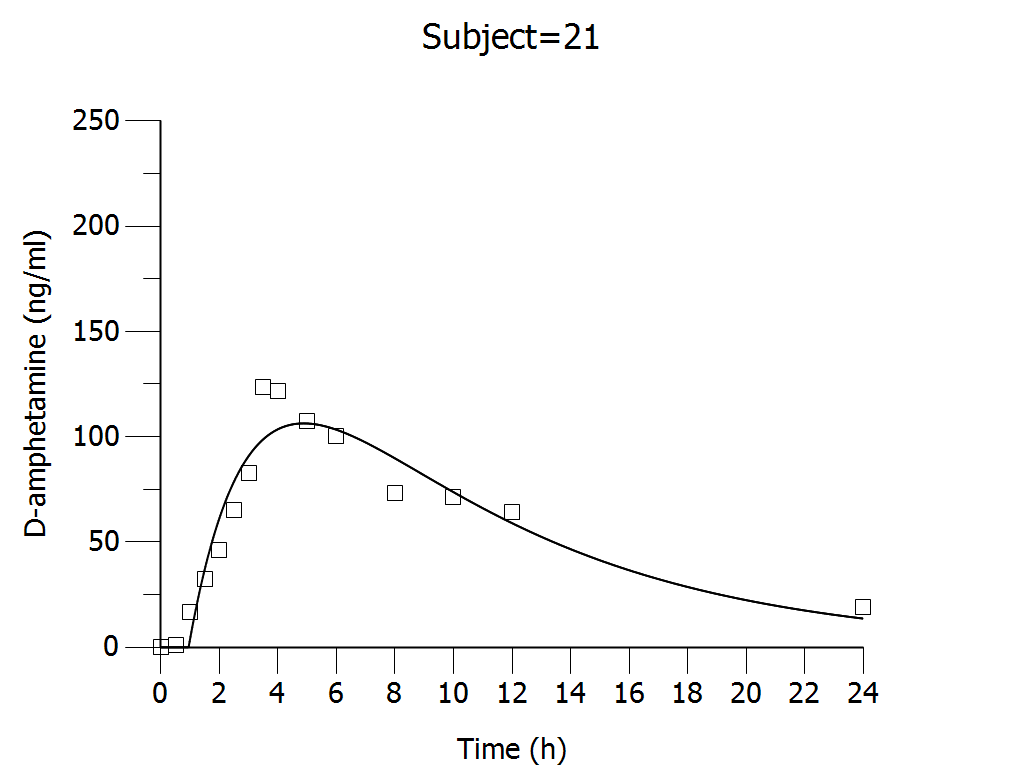 |
| 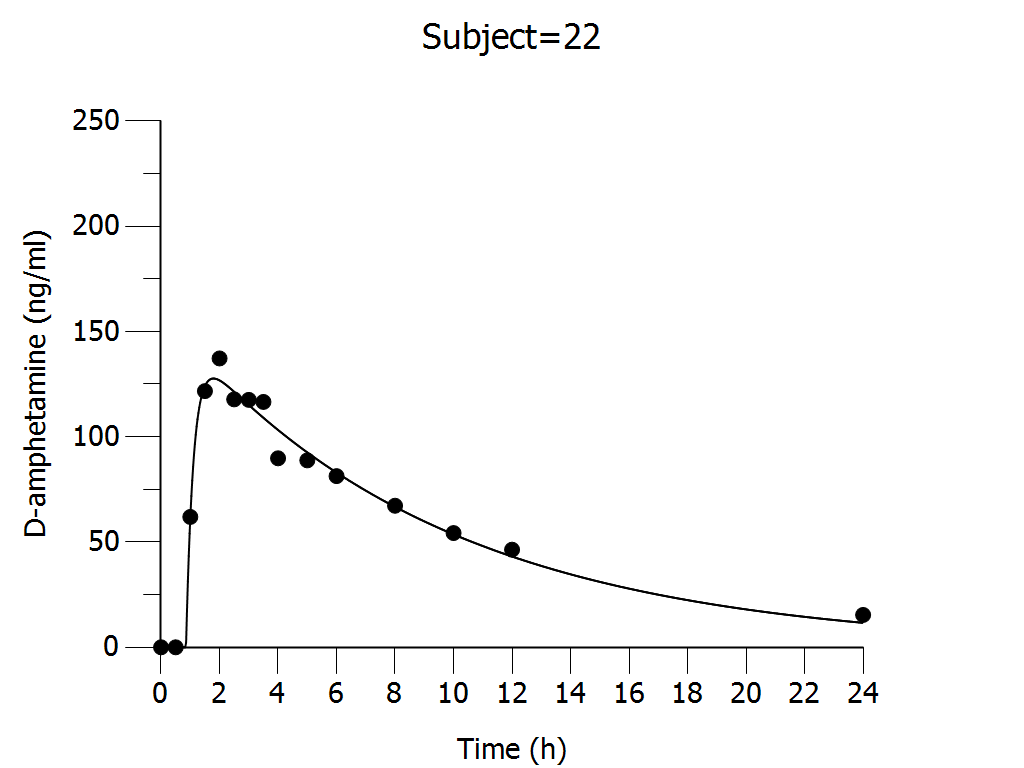 | 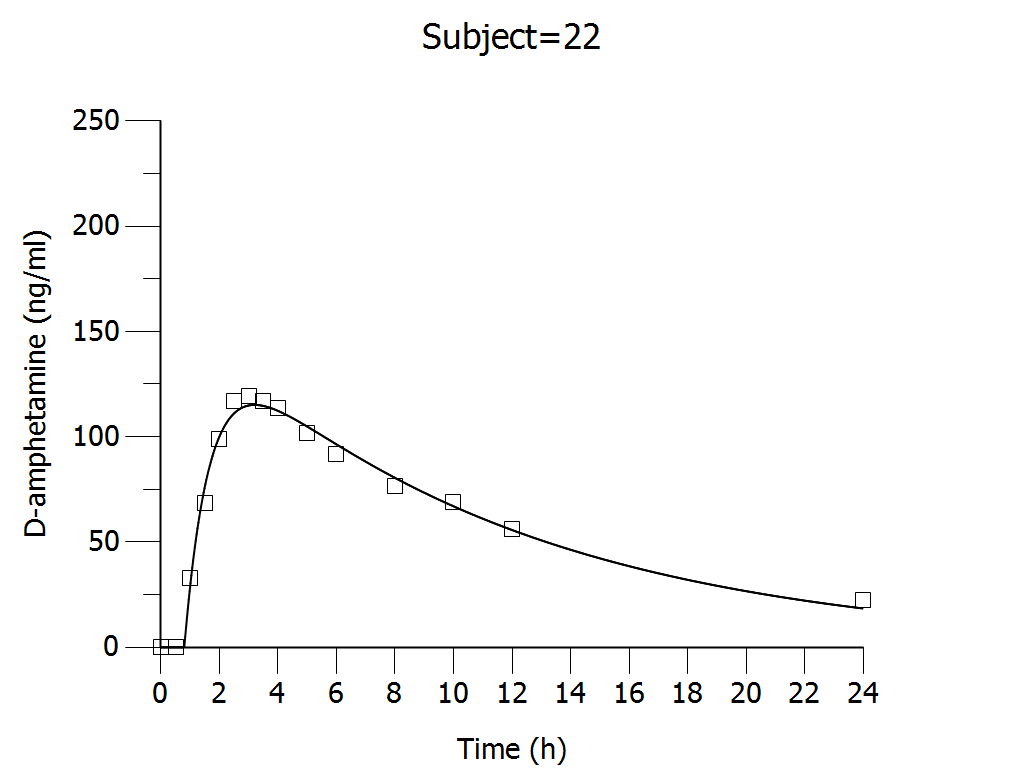 |
| 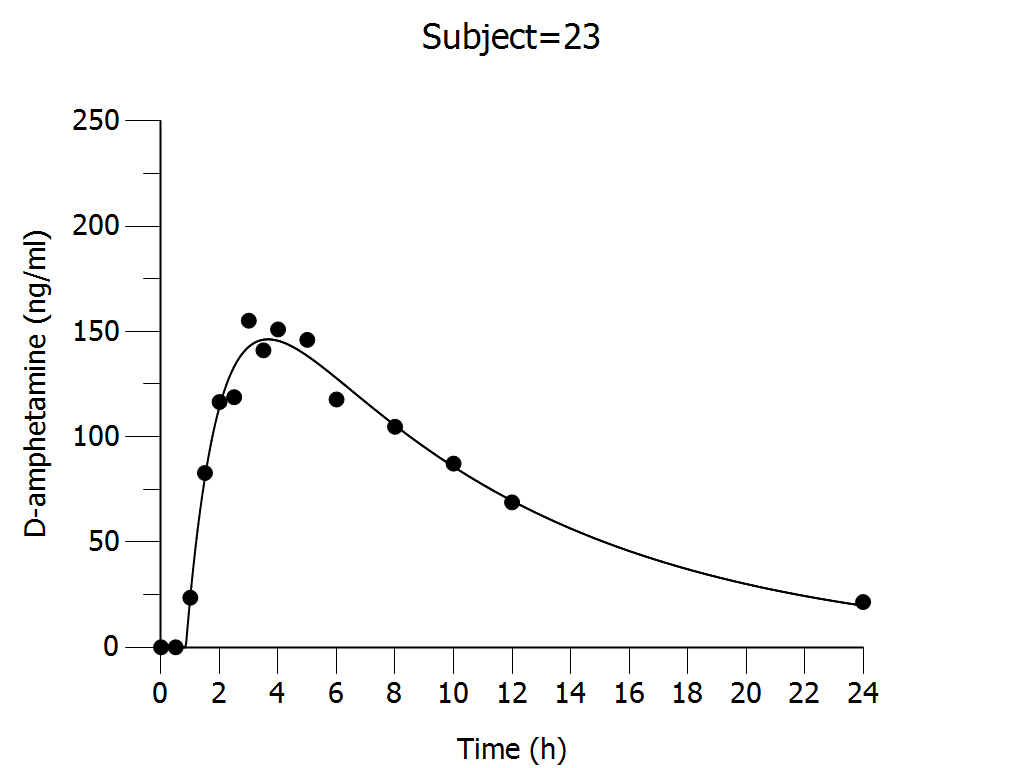 | 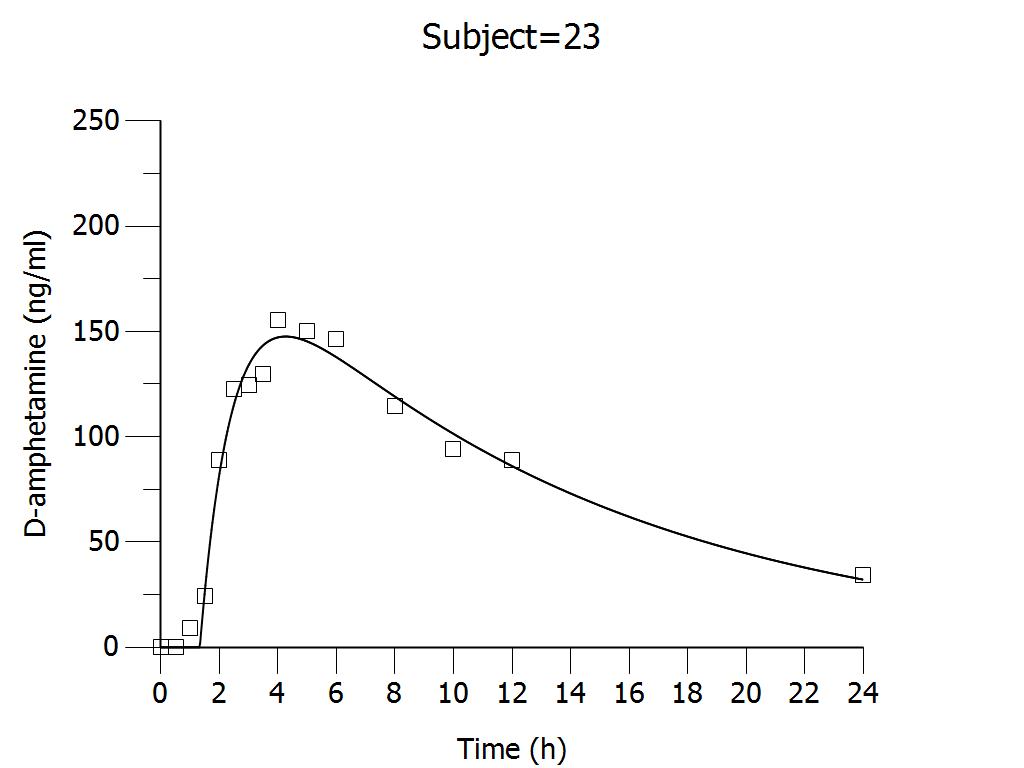 |
| **Figure S1.** Individual amphetamine plasma concentration-time curves. D-amphetamine was orally administered at a dose of 40 mg and lisdexamfetamine was administered at a dose of 100 mg at t = 0 in the same subjects. The data represent individual observed amphetamine plasma concentrations as measured at the different time points (● for D-amphetamine and □ for lisdexamfetamine) and the amphetamine concentrations predicted by the one-compartment pharmacokinetic model (black lines). Note the longer lag time in the lisdexamfetamine condition in most subjects but the otherwise similar curves shapes including similarly steep rates of increasing amphetamine concentrations. | 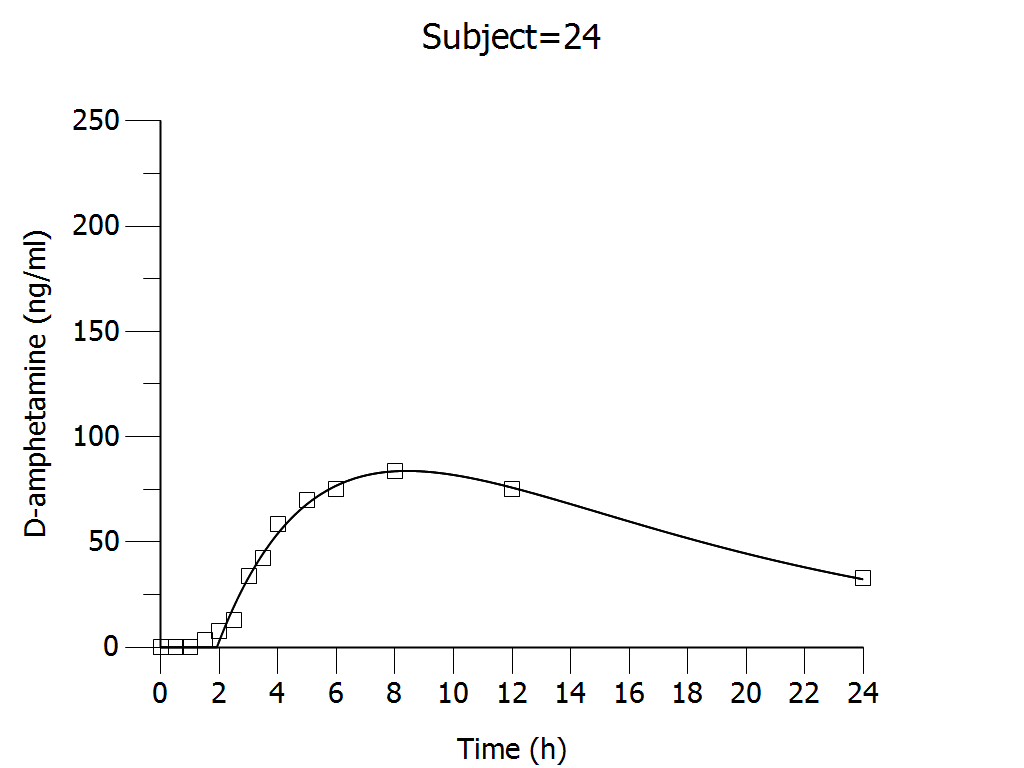 |
