# Supplementary material for: Pharmacokinetics and Pharmacodynamics of Lisdexamfetamine Compared with D-Amphetamine in Healthy Subjects
Source: Front Pharmacol. 2017 Sep 7;8:617. doi: 10.3389/fphar.2017.00617 (PMC5594082; doi:10.3389/fphar.2017.00617)
Supplement: Supplementary file 6 [file Supplementary_Methods.DOC]

**Supplemental methods**

**Quantification of amphetamine in human plasma samples**

**Chemicals and reagents:** HPLC-grade purity methanol and formic acid were purchased from Sigma-Aldrich (St. Louis, MO) or Biosolve (Dieuze, France). Distilled water was deionized using a MilliQ water purification system (Millipore, USA). Solutions of D-amphetamine hydrochloride and D-amphetamine-D_3_ sulfate >99.9% were obtained from Lipomed (Arlesheim, Switzerland). All other chemicals were purchased from Sigma-Aldrich (St. Louis, MO), and of the highest grade available.

**Instrumentation and analytical conditions**

**Analytical instruments:** Ultra-High pressure liquid chromatography-tandem mass spectrometry (UHPLC-MS/MS) using a Agilent 1290 UHPLC instrument equipped with a binary solvent delivery system, an auto sampler (at 4°C), and a column oven, coupled to an Agilent 6490 triple quadrupole mass spectrometer equipped with a jet stream electrospray ionization interface (AJS-ESI) (Agilent Technologies, Basel, Switzerland) was used to determine D-amphetamine and D-amphetamine-D_3_.

**Liquid chromatography:** The chromatographic separation was performed on a Waters Acquity UPLC BEH C18, 1.7 µm, 2.1×150 mm, column (Waters, Wexford, Ireland) at column temperature of 65ºC. The mobile phase was water-methanol-formic acid (41/59/0.1; v/v/v) and the flow rate was set at 0.45 mL/min. The analysis time was 1.5 min. A methanol in water (75/25 v/v) mixture was used as needle and needle-seat flushing solvent for 10 s after sample aspiration. Samples were stored until analysis in the auto sampler (maintained at 4°C). The injection volume was 3 µL per sample. Under these conditions, D-amphetamine and D-amphetamine-D_3_ showed a retention time of 0.8 min.

**Mass spectrometry:** Characteristic precursor ions and their corresponding product ions for multiple reaction monitoring (MRM) were defined by using the compound optimizer software module included within the Mass Hunter Workstation software (Agilent Technologies, California, USA). D-amphetamine and D-amphetamine-D_3_ (internal standard) were quantified using the corresponding mass transitions (D-amphetamine m/z 136.1→91.0 (16 V, Dwell 100 ms), m/z 136.1🡪119 (12 V, Dwell 100 ms) and D-amphetamine-D_3_ m/z 139.1→94.0 (16 V, Dwell 10 ms)). The AJS-ESI source conditions were optimized using the integrated source optimizer tool and set in the positive ion mode as following: Nitrogen gas temperature (290°C), gas flow (14 L/min), nebulizer (20 psi), sheath gas temperature (300 °C), sheath gas flow (11 L/min), capillary voltage (3000 V), and nozzle voltage (1500 V) (Agilent Technologies, California, USA, B.08.00/Build 8.0.8023.0).

**Data analysis:** The Mass Hunter Workstation Acquisition software Version B.08.00/Build 8.0.8023.0 and MassHunter Workstation Software Quantitative Analysis Version B.07.01 /Build 7.1.524.0, respectively (Agilent Technologies, California, USA) was used for data acquisition and subsequent data analysis.

**Standard solutions:** D-amphetamine hydrochloride (1 mg free base /1 mL methanol) and D-amphetamine-D_3_ sulfate (0.1 mg free base /1 mL methanol) solutions were bought as reference standards. Stock solutions in methanol containing 10 µL/mL D-amphetamine or D-amphetamine-D_3_ were prepared and stored at -20ºC.

**Sample preparation:** To 100 μL of sample, calibrator or quality control, 20 μL of a D-amphetamine-D_3_ internal standard solution (0.25 µg/mL), and 500 μL ethyl acetate for liquid–liquid extraction was added. The samples were shortly vortexed, vigorously mixed on a rotating mixer for 5 min, and centrifuged for 10 min at 16,000 x g at 4°C. The upper ethyl acetate layer (350 µL) was transferred into fresh vials and evaporated to dryness under nitrogen. Afterwards the samples were reconstituted in 50 µL methanol (10 min, 1300 rpm, 4°C, thermoshaker) and transferred into new glass vials.

**Chromatographic performance:** Ten-point calibration curves over the range of 0.78 to 200 ng/mL for D-amphetamine were generated by a zero sample and nine calibrators in human plasma. The coefficient of determination (R^2^) was 0.99 and at least 75% of all calibrators have to be valid.

**Specificity:** Human plasma samples without the addition of D-amphetamine and D-amphetamine-D_3_ were processed and injected into the UHPLC–MS/MS within an analytical run. The peak areas evaluated in the blank samples were not allowed to exceed 20% of the mean LLOQ peak area.

**Recovery:** By comparing the mean peak areas of extracted with those of unextracted samples (100% recovery) the absolute recovery was determined. The D-amphetamine recoveries were 101.7%, 102.2%, and 100.3% at concentrations of 1.66, 12.5, and 100 ng/mL.

**Limit of detection (LLOD) and limit of quantification (LLOQ):** Lower limit of detection (LLOD) and lower limit of quantification (LLOQ) were assessed by analyzing decreasing amounts of D-amphetamine in human plasma and were calculated as the concentration giving peaks with a signal-to-noise ratio of ≥ 5 and ≥ 10, respectively. The LLOQ was decided as the lowest concentration on the calibration curve which fulfilled the criteria of imprecision below 20%, and inaccuracy within ±20%. The method had a LLOD of 0.26 ng/mL, respectively a LLOQ of 0.78 ng/mL for D-amphetamine.

**Reproducibility:** Five replicates of quality controls (QCs) at the concentration of 1.66, 12.5, and 100 ng/mL were processed and injected into the UHPLC–MS/MS. To ensure the reproducibility, these sets of QCs were tested within validation runs. In each run, intra-run imprecision (% coefficient of variation; CV%) of each QC series had to be below 15% (20% at the LLOQ) and intra-run inaccuracy (% relative error of measurement; RE%) had to be within ±15% of the nominal values (±20% at the LLOQ). The intra-day precision was less than 8.8% and the accuracy ranged from −12.5 to 14.9% throughout all QC concentrations.

**Stability:** The stability of D-amphetamine in human plasma was assessed using QC at the concentrations of 1.66, 12.5, and 100 ng/ml. The samples were reanalyzed after kept at different storage conditions. The determined auto sampler stability (QC stored at 4°C for 24 h), as well as the short-term stability (storage of QC samples at −20°C for 1-week) were within ±15% of the nominal values.
